# Multi-omics analyses reveal relationships among dairy consumption, gut microbiota and cardiometabolic health

**DOI:** 10.1016/j.ebiom.2021.103284

**Published:** 2021-03-19

**Authors:** Menglei Shuai, Luo-Shi-Yuan Zuo, Zelei Miao, Wanglong Gou, Fengzhe Xu, Zengliang Jiang, Chu-wen Ling, Yuanqing Fu, Feng Xiong, Yu-ming Chen, Ju-Sheng Zheng

**Affiliations:** a School of Life Sciences, Fudan University, Shanghai, China; b Key Laboratory of Growth Regulation and Translational Research of Zhejiang Province, School of Life Sciences, Westlake University, Hangzhou, China; c Guangdong Provincial Key Laboratory of Food, Nutrition and Health; Department of Epidemiology, School of Public Health, Sun Yat-sen University, Guangzhou, China; d Institute of Basic Medical Sciences, Westlake Institute for Advanced Study, Hangzhou, China; e Westlake Laboratory of Life Sciences and Biomedicine, Hangzhou, China

**Keywords:** Dairy product, Milk, Yogurt, Gut microbiota, Metabolomics, Cardiometabolic risk factors, rRNA, ribosomal RNA, GNHS, Guangzhou Nutrition and Health Study, FFQ, food-frequency questionnaire, PCoA, principal coordinate analysis, PERMANOVA, permutational multivariate analysis of variance, LEfSe, linear discriminant analysis effect size, TC, total cholesterol, HDL, high-density lipoprotein, LDL, low-density lipoprotein, HbA1c, glycated hemoglobin

## Abstract

**Background:**

Little is known about the interplay among dairy intake, gut microbiota and cardiometabolic health in human prospective cohort studies.

**Methods:**

The present study included 1780 participants from the Guangzhou Nutrition and Health Study. We examined the prospective association between habitual dairy consumption (total dairy, milk, yogurt) and gut microbial composition using linear regression after adjusting for socio-demographic, lifestyle and dietary factors. The cross-sectional association of dairy-associated microbial features with cardiometabolic risk factors was examined with a linear regression model, adjusting for potential confounders. Serum metabolomic profiles were analyzed by partial correlation analysis.

**Findings:**

There was a significant overall difference in gut microbial community structure (β-diversity) comparing the highest with the lowest category for each of total dairy, milk and yogurt (P < 0.05). We observed that dairy-associated microbes and α-diversity indices were inversely associated with blood triglycerides, while positively associated with high-density lipoprotein cholesterol. A follow-up metabolomics analysis revealed the association of targeted serum metabolites with dairy-microbial features and cardiometabolic traits. Specifically, 2-hydroxy-3-methylbutyric acid, 2-hydroxybutyric acid and L-alanine were inversely associated with dairy-microbial score, while positively associated with triglycerides (FDR-corrected P < 0.1).

**Interpretation:**

Dairy consumption is associated with the gut microbial composition and a higher α-diversity, which provides new insights into the understanding of dairy-gut microbiota interactions and their relationship with cardiometabolic health.

**Funding:**

This work was supported by the National Natural Science Foundation of China, Zhejiang Ten-thousand Talents Program, Westlake University and the 5010 Program for Clinical Researches of the Sun Yat-sen University.

## 1 Introduction

Human gut microbiota consists of trillions of microbial cells and thousands of bacterial species. Among those factors influencing the gut microbiota, diet is a pivotal component [1,2]. Previous research showed that gut microbiota could rapidly respond to an altered diet within 1-2 days [3]. However, the long-term dietary intake seems to be the dominant force that shapes the structure and activity of the gut microbiota in humans [2,4]. While some studies have indicated the effects of dairy consumption on the gut microbiota, they are generally cross-sectional or short-term intervention studies, or only focus on several gut bacteria [5], [6], [7], [8], [9]. So far, the prospective association between long-term habitual dairy intake and gut microbial diversity and composition remains unclear.

Dairy is a diverse food group with various nutrients. However, its influence on host health cannot be characterized by individual nutrients [10]. Compared with discrete nutrients, food groups may play a more important role in the development of chronic diseases [11]. Recent large cohort studies suggested that dairy food intake was inversely associated with risk of cardiometabolic diseases, but the specific protective mechanism has yet to be identified [12], [13], [14]. On the other hand, growing evidence has revealed that the gut microbiota composition was associated with cardiometabolic diseases, such as obesity, type 2 diabetes and arterial stiffness [15], [16], [17]. Therefore, it is of great interest to investigate the relationship of dairy food-related gut microbiota structure or composition with cardiometabolic risk factors, which could help demonstrate the mechanism behind the association of dairy food intake with cardiometabolic diseases. We hypothesized that the gut microbial features and related circulating metabolites modulated by dairy food intake may contribute to the beneficial association of dairy intake with the cardiometabolic risk factors.

The primary aim of the present study was to examine the influence of habitual dairy consumption (total, milk and yogurt) on gut microbiota structure and composition in a Chinese population with relatively low dairy intake. As a secondary aim, we investigated the association of the dairy-responsive gut bacteria and derived microbial features with cardiometabolic risk factors. In addition, we investigated whether these associations might be accounted for by specific serum metabolites.

## 2 Methods

### 2.1 Study design and participants

The present study was based on the Guangzhou Nutrition and Health Study (GNHS), a community-based prospective cohort study in Guangzhou, China. Between 2008-2013, a total of 4048 participants, aged 45-70 years, who lived in Guangzhou city for at least 5 years were enrolled in the GNHS. Of these participants, 3169 (part 1) were recruited between 2008 and 2010, and 879 (part 2) were recruited between 2012 and 2013. All participants were followed up approximately every 3 years. Detailed information on the study design has been described previously [18].

For the current study, those individuals with missing baseline characteristics of age, BMI or sex (n = 5), without information of dairy intake (n = 47) at baseline, or without fecal samples (n = 2127) at follow-up visits were excluded. At baseline, we also excluded the participants with prevalent type 2 diabetes (n = 101), self-reported cancers (n = 7), chronic renal dysfunction (n = 2) or implausible dairy energy intake (< 800 or > 4000 kcal/day for men and < 500 or > 3500 kcal/day for women) (n = 16). In addition, participants who used antibiotics within two weeks before the collection of fecal samples were excluded (n = 17). Therefore, 1780 participants were finally included in this analysis (Supplemental Fig S1), with a median follow-up of 6.2 years from the baseline visit to the collection of stool samples. The study protocol was approved by the Ethics Committee of the School of Public Health at Sun Yat-sen University (2018048) and Ethics Committee of Westlake University (20190114ZJS0003). All participants provided written informed consent.

### 2.2 Data collection

Data on demographics, lifestyle, medical history and physical activity were collected by questionnaires. Education attainment was categorized into primary (0-6 years), secondary (7-9 years) and higher education (≥ 10 years). Smoking status was categorized into current smoker and non-smoker. Alcohol drinking was classified as current drinker and non-drinker. Physical activity was assessed as total metabolic equivalent for task (MET) hours per day based on a validated physical activity questionnaire [19]. Body weight, height, waist circumference, and blood pressure were measured by trained nurses on site.

Fasting venous blood samples were collected at both baseline and follow-up visits. Glucose, total triglycerides, high-density lipoprotein (HDL) cholesterol, low-density lipoprotein (LDL) cholesterol, and total cholesterol in serum were measured on an automated analyser (Roche cobas 8000 c702, Shanghai, China). Glycated hemoglobin (HbA1c) was measured with the Bole D-10 Hemoglobin A1c Program on a Bole D-10 Hemoglobin Testing System.

Habitual dietary intakes were estimated from a validated food frequency questionnaire (FFQ), which recorded the frequencies of foods consumed by the participants in the past 12 months [20]. Total dairy includes various types of milk products (whole milk, skimmed milk, whole milk powder, and skimmed milk powder) and yogurt. Milk products consumed by the cohort participants are almost exclusively regular dairy without fermentation, except for yogurt, which is fermented. We computed standard serving size for total dairy and the following subtypes of dairy products: whole milk, skimmed milk, whole milk powder, skimmed milk powder, and yogurt. The amount per serving for each dairy food was 250 g for whole milk and skimmed milk, 250 g for yogurt, 40 g for whole milk powder and skimmed milk powder [21]. Because cheese consumption in our cohort was very low/minimal, it was not included in the analysis.

### 2.3 Gut microbiota data profiling

Fecal samples of 1780 participants were collected at a follow-up visit. Microbial DNA was extracted from each sample using the QIAamp DNA Stool Mini Kit (Qiagen, Hilden, Germany) per the manufacturer's instruction. The V3-V4 hypervariable region of the 16S rRNA gene was amplified from extracted genomic DNA using primers 341F(CCTACGGGNGGCWGCAG) and 805R(GACTACHVGGGTATCTAATCC). MiSeq Reagent Kits v2 (Illumina Inc.) was used to perform amplicon sequencing on the Illumina MiSeq System (Illumina Inc., CA, USA), which generated 2  ×  250 bp paired-end sequencing data with dual-index reads.

Raw data were demultiplexed by the MiSeq Controller Software (Illumina Inc.). The sequences were trimmed for amplification primers, diversity spacers, and sequencing adapters, merge-paired and quality filtered by USEARCH [22]. UPARSE was used for operational taxonomy units (OTUs) clustering equalling or above 97%, excluding only singleton sequences prior to clustering [23]. For the taxonomy annotation, the OTUs were aligned with the ribosomal database project (RDP) classifier [24]. A representative sequence was picked for each OTU and the Greengenes 13.8 reference database was used to annotate taxonomic information for each representative sequence. In addition, the OTUs were analyzed in the Quantitative Insights into Microbial Ecology (QIIME) software version 1.9.0 [25].

### 2.4 Targeted serum metabolome profiling

The targeted metabolomics profiling of serum samples (n = 948) at the follow up visit (the same time point as the stool collection) was performed by an ultra-performance liquid chromatography coupled to tandem mass spectrometry (UPLC-MS/MS) system. Briefly, the order of all samples was randomly selected prior to preparation. 25μl serum vortexed vigorously with 100μl contain internal standards ice methanol for five minutes. At Biomek 4000 station (Biomek 4000, Beckman Coulter, Inc., Brea, California, USA), 30μl supernatant was derived with 20μl freshly prepared derivatives and mixed with internal standards in 30°C, 60min. The derivatization agents were 200 mM 3-NPH in 75% aqueous methanol and 96 mM EDC-6% pyridine solution in methanol. After derivatization, 350μl ice-cold 50% methanol solution was added to dilute the sample and then retained at -20°C for 20 minutes. After 4000g centrifugation at 4°C for 30 minutes, 135μl supernatant was mixed and sealed with internal standards for each sample. Subsequently, the derivatized samples and serial dilutions of derivatized stock standards were analyzed randomly and quantitated by the UPLC-MS/MS. The instrument setting was: ACQUITY UPLC BEH C18 analytical column (2.1*100 mm,1.7μM); column temperature 40°C; flow rate 0.4 mL/min; mobile phases A (water with 0.1% formic acid), mobile phases B (acetonitrile: IPA, 90:10); 0-1 min (5% B), 1-12 min (5-80% B), 12-15 min (80-95% B), 15-16 min (95-100%B), 16-18 min (100%B), 18-18.1 min (100-5% B), 18.1-20 min (5% B); 1.5Kv (ESI+), 2.0Kv (ESI-) capillary.

Three types of quality control samples, i.e. test mixtures, internal standards, and pooled biological samples were used in the metabolomics platform. The internal standards (172 isotope-labeled compounds) were added to the test samples in order to monitor analytical variations during the entire sample preparation and analysis process. The derivatized pooled samples for quality control were injected per 14 samples. Raw data generated by UPLC-MS/MS were processed using the QuanMET software (v2.0, Metabo-Profile, Co., Ltd, Shanghai, China) to perform peak integration, calibration, and quantification for each metabolite. The list of metabolites was selected to capture the microbiota-related metabolites and some key host metabolites. Finally, 199 metabolites were selected. These metabolites mainly include amino acids, bile acids, fatty acids, carbohydrates, organic acids, nucleosides, indoles, benzenoids, phenylpropanoic acids, pyridines, and carnitines.

### 2.5 Statistical analysis

Statistical analysis was performed using Stata version 15 and R version 3.5.3. We used a linear mixed model to examine the association of sequencing depth and sequencing run (as a random effect) with z-score normalized α-diversity indices (Shannon, Simpson and Observed species). Residuals of the model were taken as technique-adjusted α-diversity indices for subsequent analysis.

Participants were categorized into different groups based on their dairy consumption (total dairy and milk: <1serving/month, 1 serving/month-1 serving/week, 1 serving/week-0.5 serving/day, ≥0.5 serving/day; as for yogurt, the highest group was ≥1 serving/week). 10000 randomly chosen 16S rRNA sequences were used to calculate α-diversity indices (Observed species, Shannon index, Simpson index). We examined the prospective association between each of the dairy categorical variables (total dairy, milk and yogurt) and α-diversity indices using a linear regression model, adjusted for potential confounders as follows: model 1 - baseline age and sex; model 2 – as model 1 plus baseline body mass index (BMI), total energy intake, physical activity, smoking status, drinking status, education attainment and household income level; model 3 – as model 2 plus baseline dietary intakes of vegetable, fruit, fish, egg and red meat. We also used the same models to assess the linear trend of the above associations by assigning the median value of intake to each category. Principal coordinate analysis (PCoA) based on Bray-Curtis distance and permutational multivariate analysis of variance (PERMANOVA) (999 permutations) were performed to examine the association between gut microbial community structure and dairy intake using the vegan R package (v2.5-6) [26]. We compared the β-diversity dissimilarities between the highest and the lowest dairy consumption category at OTUs levels. At genus level, we applied LEfSe (Linear discriminant analysis Effect Size) with default parameters (α value for Wilcoxon tests:0.05; the logarithmic LDA score threshold:2.0) to identify specific gut microbes associated with dairy intakes [27]. As we only used the dairy intake information at baseline of the cohort, it might be that the dairy intake would change over time. To address this concern, we calculated the intraclass correlation coefficients (ICC) for the dairy variables using FFQ data collected at both two time points (n = 1098).

Subsequently, we generated a dairy-microbial score as a new gut microbial feature to represent the gut bacteria group associated with total dairy intake or different subtypes. Based on the relative abundance of identified biomarkers (genus level) and LDA score from LEfSe analysis, we used the below formula to compute dairy-microbial scores (dairy-microbial score = ∑ (+/– relative abundance of biomarkers of genus; here +/– depends on LDA score, if LDA score > 0, it is assigned a “+”, otherwise it is assigned a “–”). To test the reliability of these scores, we performed a linear regression analysis to investigate whether the scores were associated with each of the dairy intake variables after multivariable adjustment. In addition, we conducted analyses stratified by age (<65 vs ≥65), sex (men vs women) and body mass index (<25 vs ≥25) to assess potential effect modification.

We used a multivariable linear regression model to assess the cross-sectional association of the gut microbial features (including α-diversity indices and dairy-microbial scores) with cardiometabolic risk factors, including BMI, waist circumference, blood fasting glucose, HbA1c, triglycerides, HDL cholesterol, LDL cholesterol, total cholesterol, systolic blood pressure and diastolic blood pressure, adjusted for potential confounders. The dependent variables with skewed distribution were log-transformed before analysis (TC/HDL-C, TG, glucose and HDL cholesterol). The associations were expressed as the difference in cardiometabolic risk factors (in SD unit) per 1 SD difference in each gut microbial feature. We then used a Spearman correlation analysis to examine the correlations between dairy-related specific gut bacteria and cardiometabolic traits. The relative abundances of gut bacteria were normalized before correlation test. The Benjamini-Hochberg method was used to control the false discovery rate (FDR, 5%) for the multiple testing of the above cross-sectional analyses and Spearman correlation analyses [28].

To test the potential effect modification of gut microbiota, we used a linear regression model to examine the interaction of corresponding dairy intakes with microbial features on the cardiometabolic risk factors, adjusted for the same covariates as above cross-sectional analysis model. If a significant interaction (P < 0.05) was found, we further conducted subgroup analysis stratified by the dairy consumption categories to assess the association between the gut microbial features and the corresponding cardiometabolic trait. We also conducted subgroup analysis stratified by gut microbial diversity index to investigate the dairy-cardiometabolic trait association.

For the analysis of the potential role of serum metabolome, we first used a partial Spearman correlation analysis to identified serum metabolites associated with milk-microbial score or yogurt-microbial score, adjusted for age, sex and BMI. Then, we examined the associations of the above-identified metabolites with dairy consumption and cardiometabolic traits using the same model (FDR-corrected P < 0.1).

### 2.6 Role of the funding source

This work was supported by the National Natural Science Foundation of China (81903316, 82073529, 81773416), Zhejiang Ten-thousand Talents Program (2019R52039), Westlake University (101396021801) and the 5010 Program for Clinical Researches (2007032) of the Sun Yat-sen University (Guangzhou, China). The funders were not involved in the study design, implementation, the analysis or the interpretation of data.

## 3 Results

### 3.1 Characteristics of study population

The baseline mean value for age and BMI of 1780 participants in the present study was 58 y (SD: 6.0 y) and 23.2 kg/m^2^ (SD: 3.0 kg/m^2^), respectively (Table 1). Those individuals with higher total dairy consumption were more likely to be women, non-smokers, at a higher income level and had a higher education level (Table 1).

**Table 1:** Baseline characteristics of the study population by total dairy consumption (n = 1780) ^a^.

Characteristics	Total dairy consumption (servings)			
	<1/mo	1/mo-1/wk	1/wk-0.5/d	≥0.5/d
No. participants	216	253	540	771
Age, y	58.8 (6.4)	58.4 (6.3)	58.1 (5.7)	58.5 (5.9)
Sex, n (%)				
Female	114 (52.8%)	150 (59.3%)	381 (70.6%)	561 (72.8%)
BMI^b^, kg/m^2^	23.5 (3.0)	23.4 (2.9)	23.0 (3.1)	23.1 (2.8)
Education, n (%)				
Primary	79 (36.6%)	70 (27.7%)	153 (28.3%)	180 (23.3%)
Secondary	92 (42.6%)	122 (48.2%)	262 (48.5%)	346 (44.9%)
Higher education	45 (20.8%)	61 (24.1%)	125 (23.1%)	245 (31.8%)
Income level, n (%)				
≤ 500 ¥/mo	7 (3.2%)	5 (2.0%)	7 (1.3%)	7 (0.9%)
501-1500 ¥/mo	48 (22.2%)	62 (24.5%)	120 (22.2%)	163 (21.1%)
1501-3000 ¥/mo	142 (65.7%)	159 (62.8%)	335 (62.0%)	481 (62.4%)
> 3001 ¥/mo	19 (8.8%)	27 (10.7%)	78 (14.4%)	120 (15.6%)
Smoking status, n (%)				
Nonsmoker	156 (72.2%)	200 (79.1%)	460 (85.2%)	696 (90.3%)
Smoker	60 (27.8%)	53 (20.9%)	80 (14.8%)	75 (9.7%)
Alcohol drinking, n (%)				
Nondrinker	200 (92.6%)	223 (88.1%)	508 (94.1%)	724 (93.9%)
Drinker	16 (7.4%)	30 (11.9%)	32 (5.9%)	47 (6.1%)
Physical activity^c^, n (%)				
Inactive	64 (29.6%)	68 (26.9%)	132 (24.4%)	181 (23.5%)
Moderately inactive	47 (21.8%)	65 (25.7%)	133 (24.6%)	200 (25.9%)
Moderately active	57 (26.4%)	59 (23.3%)	137 (25.4%)	192 (24.9%)
Active	48 (22.2%)	61 (24.1%)	138 (25.6%)	198 (25.7%)
Total energy intake, kcal/d	1682 (412)	1669 (534)	1762 (498)	1789 (473)
Vegetable intake, g/day	352.8 (170.2)	367.3 (351.8)	373.9 (157.2)	377.9 (176.0)
Fish intake, g/day	51.9 (81.1)	49.3 (38.6)	51.6 (46.9)	50.4 (49.2)
Red meat intake, g/day	87.4 (58.0)	83.0 (50.0)	83.8 (53.0)	79.5 (49.2)
Egg intake, g/day	28.6 (29.4)	26.7 (20.5)	30.8 (34.4)	31.7 (20.7)
Fruit intake, g/day	119.9 (111.0)	119.8 (95.0)	152.6 (112.8)	159.0 (107.9)

^a^Data are mean (SD) unless otherwise indicated.

^b^Body mass Index.

^c^Physical activity was classified into four groups according to quartiles.

### 3.2 Dairy consumption and gut microbiota composition

Total dairy consumption was positively associated with Shannon and Simpson index after adjusting for socio-demographic and lifestyle factors (model 2, both P-trend = 0.04, Table 2). The association was attenuated after further adjustment for dietary intakes (model 3, P-trend = 0.07 and 0.08 respectively, Table 2). However, participants in the highest (≥ 0.5 serving/d) and second highest (1 serving/wk-0.5 serving/d) total dairy intake category, compared with lowest category (< 1 serving/mo), showed higher levels of Shannon and Simpson index across all three statistical models (P < 0.05, Table 2). Moreover, yogurt consumption was positively associated with Shannon and Observed species after multivariable adjustment in model 2 or model 3 (P-trend < 0.05). There was no significant association between milk intake and any α-diversity index.

**Table 2:** Prospective association between dairy intake and α-diversity indices (n = 1780) ^a^.

	Group 1	Group 2	Group 3	Group 4	P-trend
Total dairy					
Groups, servings	<1/mo	1/mo-1/wk	1/wk-0.5/d	≥0.5/d	
Dairy intake^b^	0.01 (0.002-0.02)	0.08 (0.05-0.011)	0.31 (0.23-0.41)	0.81 (0.62-1.02)	
Participants	216	253	540	771	
Shannon Index					
Model 1	Reference	0.11 (-0.06, 0.27)	0.17 (0.02, 0.31)	0.19 (0.05, 0.33)	0.03
Model 2	Reference	0.10 (-0.06, 0.27)	0.16 (0.01, 0.31)	0.18 (0.04, 0.33)	0.04
Model 3	Reference	0.11 (-0.06, 0.28)	0.16 (0.01, 0.31)	0.17 (0.03, 0.32)	0.07
Simpson Index					
Model 1	Reference	0.20 (0.03, 0.38)	0.20 (0.05, 0.35)	0.23 (0.08, 0.37)	0.04
Model 2	Reference	0.20 (0.03, 0.38)	0.20 (0.04, 0.35)	0.23 (0.08, 0.38)	0.04
Model 3	Reference	0.21 (0.04, 0.38)	0.20 (0.04, 0.35)	0.22 (0.07, 0.37)	0.08
Observed Species					
Model 1	Reference	0.07 (-0.1, 0.23)	0.15 (0.01, 0.3)	0.14 (-0.001, 0.27)	0.14
Model 2	Reference	0.07 (-0.1, 0.23)	0.15 (0.005, 0.29)	0.14 (-0.003, 0.28)	0.15
Model 3	Reference	0.07 (-0.1, 0.23)	0.15 (0.001, 0.29)	0.13 (-0.02, 0.27)	0.23
Milk					
Groups	<1/mo	1/mo-1/wk	1/wk-0.5/d	≥0.5/d	
Dairy intake	0.004 (0.001-0.01)	0.08 (0.05-0.11)	0.31 (0.23-0.42)	0.84 (0.63-1.00)	
Participants	405	279	561	535	
Shannon Index					
Model 1	Reference	0.02 (-0.13, 0.16)	0.07 (-0.05, 0.19)	0.11 (-0.01, 0.23)	0.07
Model 2	Reference	0.02 (-0.13, 0.16)	0.07 (-0.05, 0.19)	0.11 (-0.02, 0.23)	0.08
Model 3	Reference	0.03 (-0.12, 0.17)	0.07 (-0.05, 0.19)	0.10 (-0.03, 0.22)	0.13
Simpson Index					
Model 1	Reference	0.06 (-0.09, 0.2)	0.06 (-0.07, 0.18)	0.12 (0.002, 0.25)	0.06
Model 2	Reference	0.06 (-0.08, 0.21)	0.06 (-0.06, 0.19)	0.13 (0.004, 0.25)	0.06
Model 3	Reference	0.07 (-0.07, 0.22)	0.06 (-0.06, 0.19)	0.12 (-0.01, 0.24)	0.10
Observed Species					
Model 1	Reference	0.01 (-0.12, 0.15)	0.05 (-0.07, 0.16)	0.09 (-0.03, 0.2)	0.13
Model 2	Reference	0.02 (-0.12, 0.15)	0.05 (-0.07, 0.17)	0.09 (-0.03, 0.21)	0.13
Model 3	Reference	0.02 (-0.11, 0.16)	0.05 (-0.07, 0.16)	0.08 (-0.04, 0.2)	0.20
Yogurt					
Groups	<1/mo	1/mo-1/wk	≥1/wk	-	
Dairy intake	0.003 (0.001-0.01)	0.08 (0.05-0.10)	0.28 (0.20-0.48)	-	
Participants	925	380	475	-	
Shannon Index					
Model 1	Reference	0.05 (-0.06, 0.16)	0.14 (0.03, 0.24)	-	0.01
Model 2	Reference	0.05 (-0.06, 0.16)	0.13 (0.02, 0.23)	-	0.02
Model 3	Reference	0.05 (-0.07, 0.16)	0.13 (0.02, 0.23)	-	0.02
Simpson Index					
Model 1	Reference	0.07 (-0.04, 0.19)	0.10 (-0.01, 0.21)	-	0.08
Model 2	Reference	0.07 (-0.04, 0.19)	0.09 (-0.02, 0.2)	-	0.12
Model 3	Reference	0.07 (-0.05, 0.18)	0.09 (-0.02, 0.2)	-	0.14
Observed Species					
Model 1	Reference	0.05 (-0.06, 0.16)	0.15 (0.05, 0.25)	-	0.004
Model 2	Reference	0.05 (-0.06, 0.16)	0.15 (0.04, 0.25)	-	0.001
Model 3	Reference	0.05 (-0.06, 0.16)	0.14 (0.04, 0.25)	-	0.01

^a^The results are presented as the change (95%CI) in each α-diversity index (in SD unit) comparing each category of dairy intake with the lowest intake group (reference, <1/mo). Beta (95% CI) and P-trend were calculated by using a linear regression model, adjusted for the following covariates: model 1, age and sex; model 2, model 1 plus BMI, total energy intake, physical activity, smoking status, drinking status, education attainment and household income level; and model 3, model 2 plus dietary intakes of vegetable, fruit, fish, egg and red meat. P-trend was assessed based on the median value of intake for each category.

^b^Data are median (IQR).

**Fig 1:**
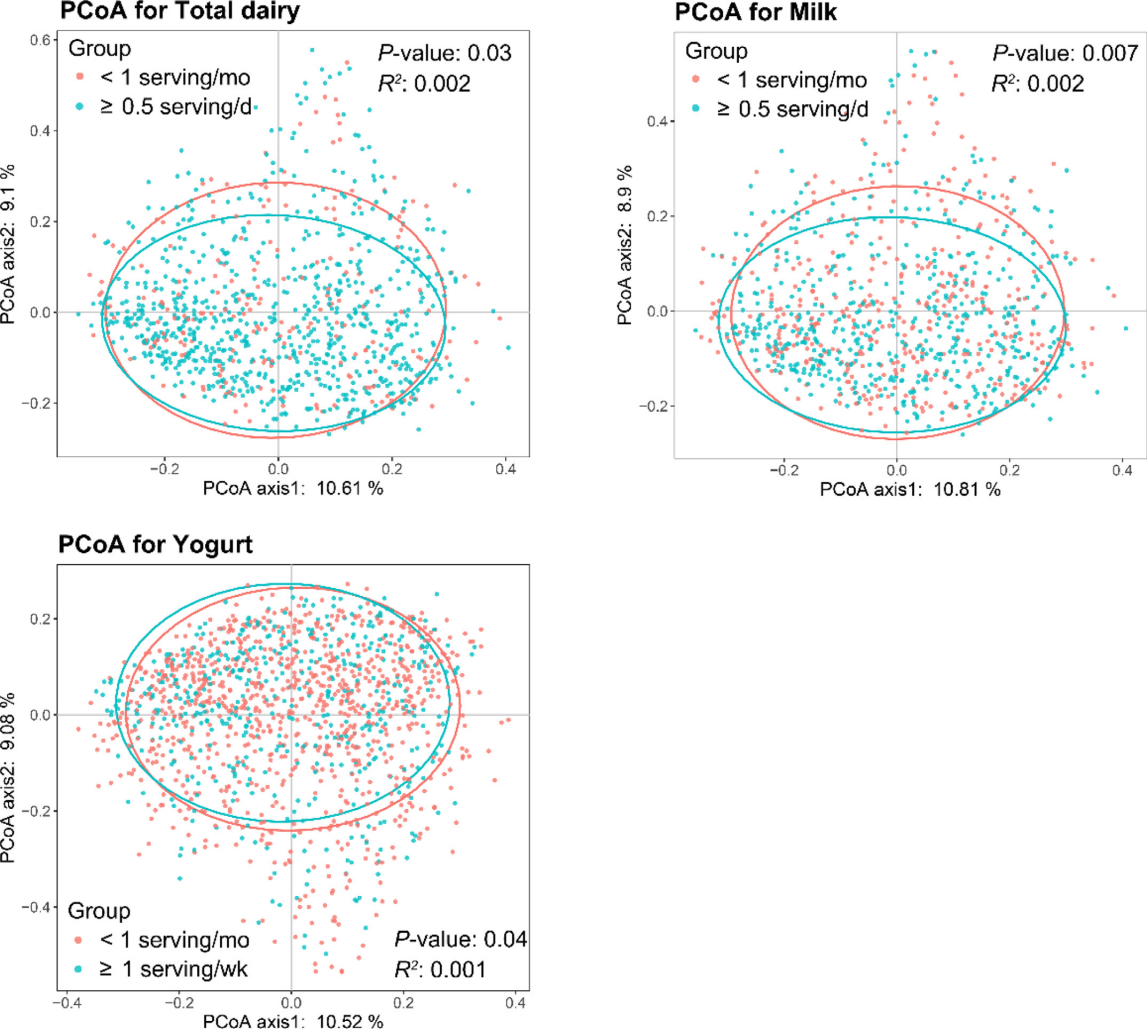
Variation of gut microbial community structure represented by PCoA plots based on Bray-Curtis distance. P value was calculated by PERMANOVA based on the participants categorized by consumption levels of total dairy, milk or yogurt. Each point represents an individual from the study. Number of participants in the high and low intake group was 771 and 216 for total dairy intake, 535 and 405 for milk intake, 475 and 925 for yogurt intake, respectively. PCoA, principle coordinate analysis.

At OTU level, we observed significant overall differences in gut microbial community structure between the highest and the lowest intake category of all three examined dairy variables using PCoA and PERMANOVA analyses (all P-values < 0.05, Fig 1).

### 3.3 Taxonomy biomarkers of long-term dairy consumption

**Fig 2:**
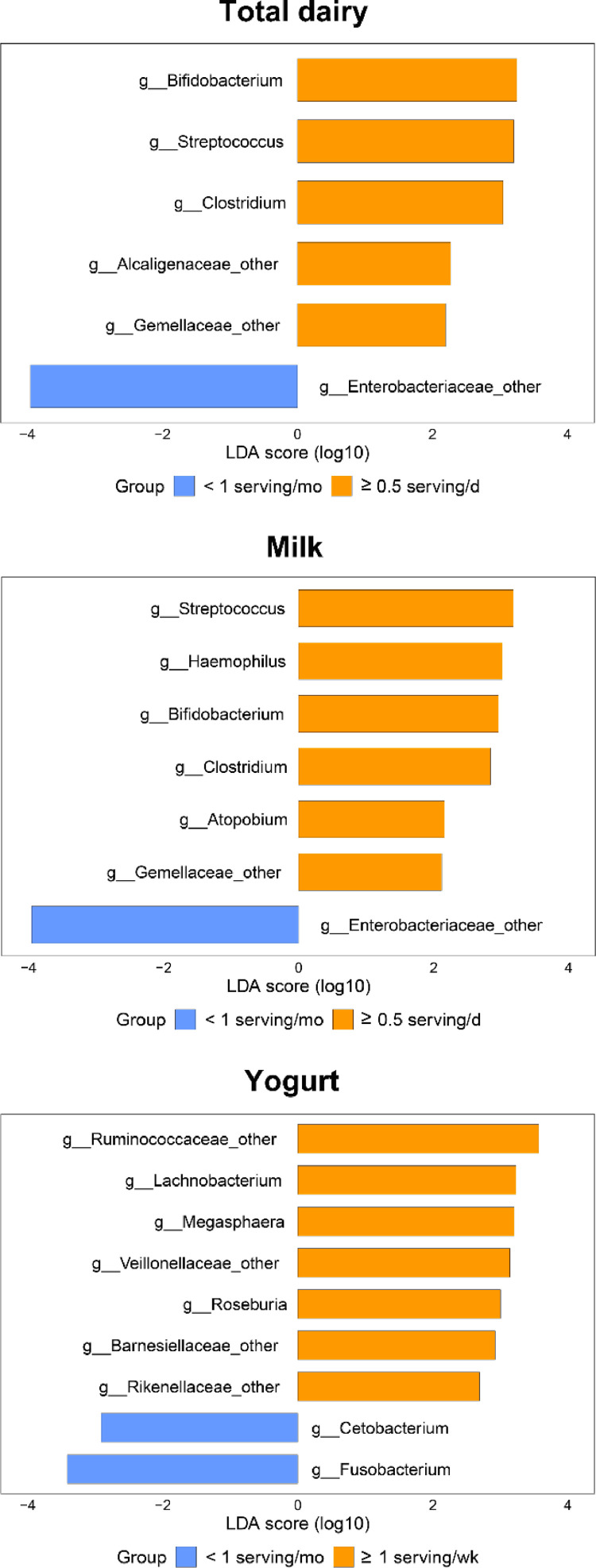
Taxonomic biomarkers associated with total dairy, milk and yogurt consumption. These biomarkers at genus level were identified by linear discriminant analysis (LEfSe). Color indicates the group in which a differentially abundant taxon is enriched (orange: higher dairy consumption; blue: lower dairy consumption). Number of participants in the high and low intake group was 771 and 216 for total dairy intake, 535 and 405 for milk intake, 475 and 925 for yogurt intake, respectively.

Our results suggested that multiple genera were enriched in the highest total dairy intake group and the highest milk intake group, respectively (Fig 2). Among those genera, Bifidobacterium, Streptococcus, Clostridium and Gemellaceae_other were enriched in both dairy groups. In contrast, a genus from family Enterobacteriaceae was identified as biomarker of lower total dairy intake and lower milk intake. Moreover, 7 genera: Ruminococcaceae_other, Lachnobacterium, Megasphaera, Veillonellaceae_other, Roseburia, Barnesiellaceae_other and Rikenellaceae_other were enriched in the highest yogurt intake group, whereas genus Cetobacterium and Fusobacterium were markers of lower yogurt consumption.

The dairy intake was relatively stable overtime, and the ICC for the total dairy, milk and yogurt intake between the baseline and follow-up visit was 0.54, 0.48, and 0.56, respectively.

We observed a significant association (P < 0.01) between the dairy variable and its corresponding dairy-microbial score generated from bacteria biomarkers after multivariable adjustment based on 16S data (Supplemental Table S1).

### 3.4 Dairy-related gut microbial features and cardiometabolic risk factors

**Fig 3:**
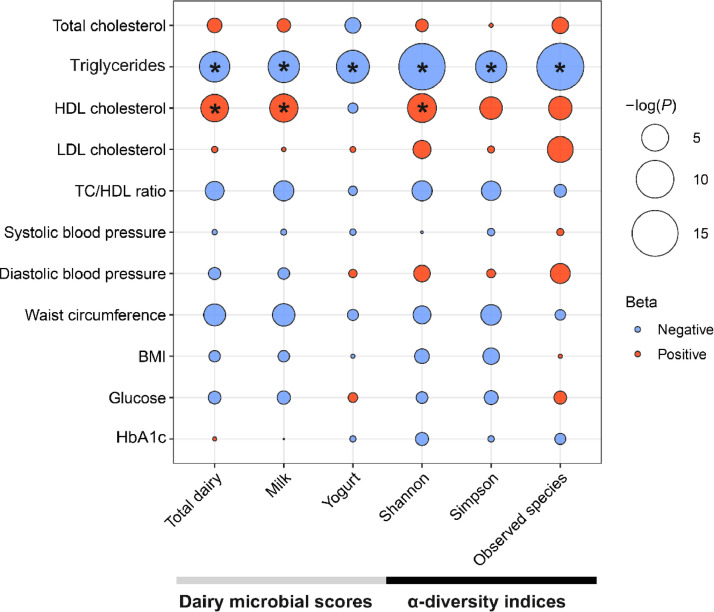
Associations between dairy-related gut microbial features and cardiometabolic risk factors. Linear regression models were adjusted for age, sex, BMI, smoking status, drinking status, education attainment, household income, physical activity, and total energy intake, dietary intakes of vegetables, fruit, fish, egg and red meat. Total number of participants in each analysis was 1713 for HDL cholesterol, LDL cholesterol, triglycerides and total cholesterol, 1767 for diastolic blood pressure, systolic blood pressure and BMI, 1712 for glucose, 1754 for waist circumference, and 1090 for HbA1c. HDL, high-density lipoprotein. LDL, low-density lipoprotein. BMI, body mass index. HbA1c, glycated hemoglobin. Triglycerides, HDL cholesterol, TC/HDL ratio and Glucose were log-transformed. *FDR-corrected P < 0.05 (Benjamini-Hochberg method).

The multivariable linear regression analysis indicated that per 1-SD increment in each of the dairy-microbial scores and α-diversity indices was associated with a 0.08-0.14 SD lower levels of serum triglycerides (P-value < 0.05 after correction of multiple testing for various cardiometabolic traits) (Fig 3, Supplemental Table S2). We also observed a positive association of total dairy-microbial score, milk-microbial score and Shannon index with HDL cholesterol (Fig 3). Moreover, the associations between dairy intake and corresponding dairy-microbial score did not significantly vary by age (<65 vs ≥65), sex or body mass index (<25 vs ≥25).

**Fig 4:**
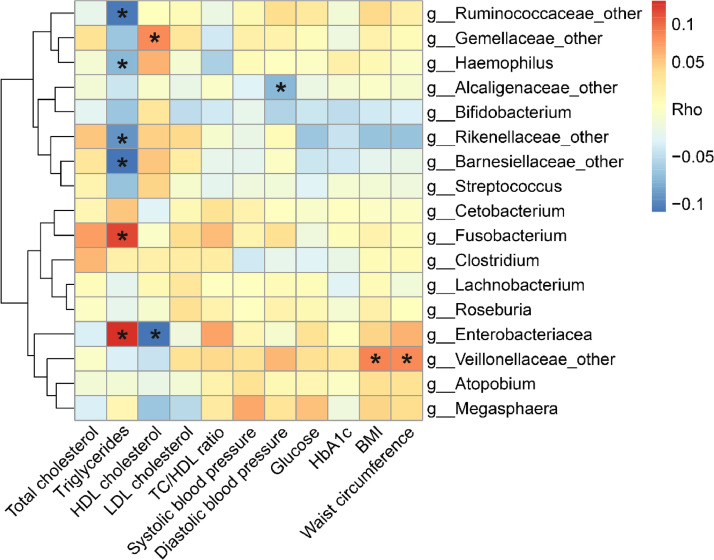
Association of dairy-related bacterial genera with cardiometabolic risk factors. The heat-map shows the Spearman correlation coefficients between individual genera and cardiometabolic risk factors. The relative abundances of genera were normalized. Triglycerides, high-density lipoprotein (HDL) cholesterol, total cholesterol/high-density lipoprotein (TC/HDL) cholesterol ratio and glucose were log-transformed. P value was corrected for multiple testing using the Benjamini-Hochberg false discovery rate. *FDR-corrected P < 0.05.

The correlation analysis showed that triglycerides was negatively correlated with the genera Ruminococcaceae_other, Haemophilus, Barnesiellaceae_other and Rikenellaceae_other, while positively associated with the genera Cetobacterium and Fusobacterium (Fig 4). In addition, HDL cholesterol was inversely correlated with the genus Enterobacteriaceae_other, which was enriched in lower total dairy and milk consumption category.

### 3.5 Interaction of dairy intake with gut microbiota on cardiometabolic trait

We observed significant interactions with gut microbiota for total dairy consumption on serum triglycerides (P for interaction < 0.05). Per 1-SD difference in Shannon index [beta = -0.08, 95% CI: (-0.11, -0.05)] and Observed species [beta = -0.09, 95% CI: (-0.12, -0.06)] was associated with lower levels of triglycerides (in SD unit) only among individuals with higher total dairy intake (≥ 1 serving/wk), but not among lower total dairy intake participants (Table 3). Similarly, in the participants with lower milk consumption (< 1 serving/wk), there was no significant association with triglycerides for Shannon index or Observed species, while in the higher milk consumption group, there was an inverse association of Shannon index and Observed species with triglycerides (P < 0.05). Yogurt-microbial score and Observed species were negatively associated with triglycerides only among higher yogurt intake participants [beta = -0.07, 95% CI: (-0.11, -0.04); beta = -0.09, 95% CI: (-0.13, -0.05)], but not among participants with lower yogurt intake (P for interaction = 0.02 and 0.04, respectively) (Table 3). On the other hand, we found that per 1 serving/d higher in total dairy and milk consumption was associated with 0.12 and 0.19 SD lower triglyceride levels, respectively, only among individuals with higher α-diversity (P < 0.05 for each of the three α-diversity indices, Supplemental Fig S2).

**Table 3:** Subgroup analysis for the association of gut microbial features with cardiometabolic risk factors by different levels of dairy consumption^a^.

Gut microbial features	Cardiometabolic trait	Subgroup	N	Beta(95% CI)	P	P for interaction
Total dairy intake						
Shannon index	Triglycerides	< 1 serving/wk	442	-0.03 (-0.08, 0.02)	0.2	0.022
		≥ 1 serving/wk	1271	-0.08 (-0.11, -0.05)	<0.001	
Observed species	Triglycerides	< 1 serving/wk	442	-0.03 (-0.08, 0.02)	0.29	0.009
		≥ 1 serving/wk	1271	-0.09 (-0.12, -0.06)	<0.001	
Milk intake						
Shannon index	Triglycerides	< 1 serving/wk	649	-0.03 (-0.07, 0.01)	0.13	0.011
		≥ 1 serving/wk	1064	-0.09 (-0.12, -0.06)	<0.001	
Observed species	Triglycerides	< 1 serving/wk	649	-0.04 (-0.08, 0.00)	0.051	0.039
		≥ 1 serving/wk	1064	-0.09 (-0.12, -0.06)	<0.001	
Yogurt intake						
Observed species	Triglycerides	< 1 serving/mo	889	-0.04 (-0.08, -0.01)	0.01	0.039
		≥ 1 serving/mo	824	-0.09 (-0.13, -0.05)	<0.001	
Yogurt-microbial score	Triglycerides	< 1 serving/mo	889	-0.01 (-0.05, 0.02)	0.37	0.019
		≥ 1 serving/mo	824	-0.07 (-0.11, -0.04)	<0.001	

a Associations were expressed as the difference in cardiometabolic risk factors (in SD unit) per 1 SD difference in each gut microbial feature. Beta (95% CI) and P were calculated in a linear regression model after adjustment for age, sex, BMI, smoking status, drinking status, education attainment, household income, physical activity, and total energy intake, dietary intakes of vegetables, fruit, fish, egg and red meat.

### 3.6 Metabolites associated with dairy-microbial scores and cardiometabolic traits

**Fig 5:**
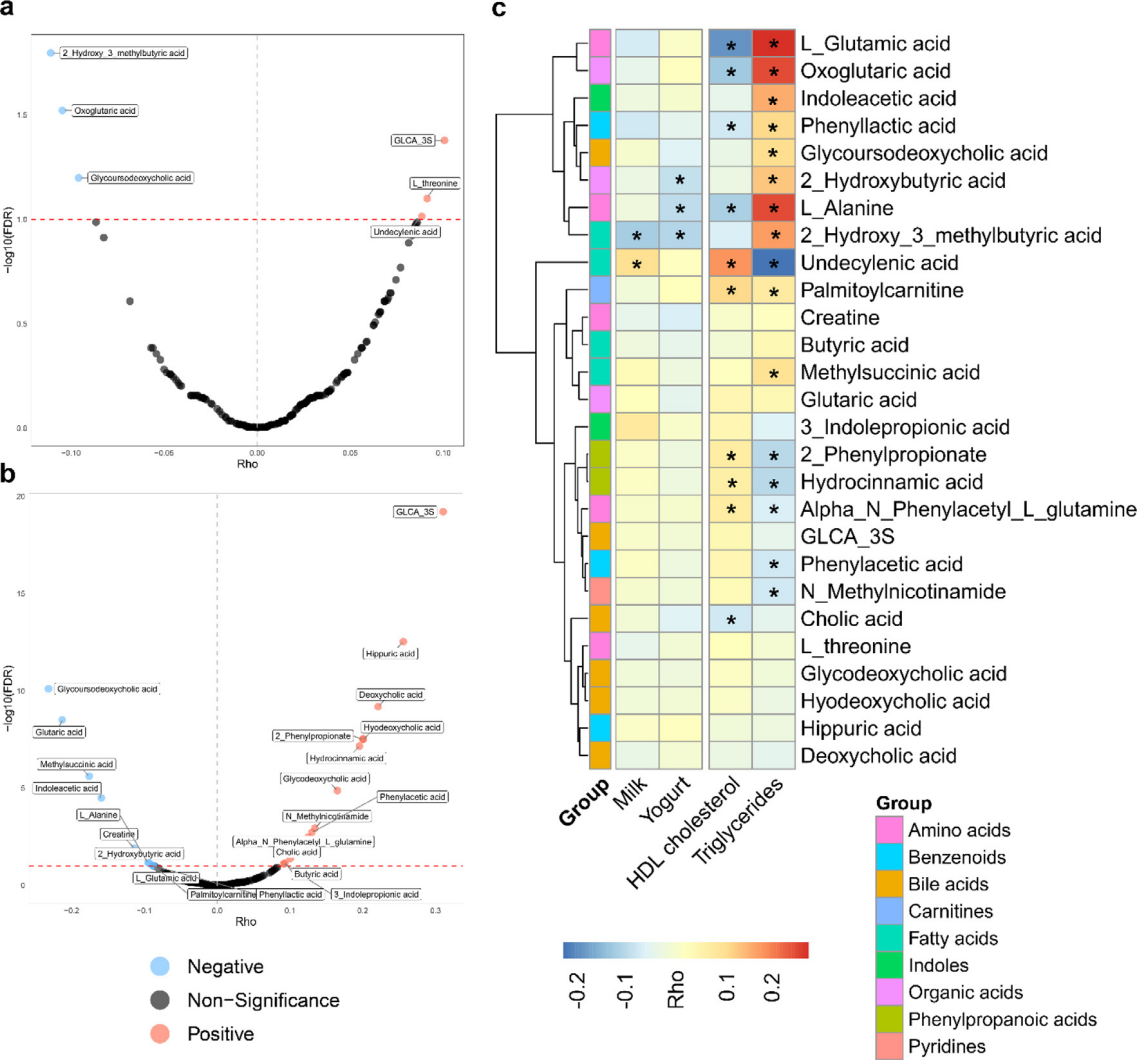
Serum metabolites linking dairy consumption, dairy-microbial scores and cardiometabolic traits. (a) The volcano plot shows the serum metabolites associated with milk-microbial score. The partial correlation analysis was adjusted for age, sex and BMI. The x axis shows the rho values and the y axis indicates the –log (base 10) of the FDR-corrected P values. Red dash lines indicate the threshold of FDR-corrected P = 0.1. Most negatively associated metabolites are expected to be at the extreme left of the plot (blue point), while the most positively associated metabolites are expected to be at the extreme right of the plot (red point). Points are colored based on the significance of the obtained associations (red and blue indicates associations with FDR-corrected P < 0.1). (b) The volcano plot shows the serum metabolites associated with yogurt-microbial score. (c) The heat map shows the associations between serum metabolites and dairy consumption and cardiometabolic traits. *FDR-corrected P < 0.05 (Benjamini-Hochberg method). Total number of participants with serum metabolomics data in the above analyses was 948. (For interpretation of the references to colour in this figure legend, the reader is referred to the web version of this article.)

Using partial Spearman correlation analysis, we identified 6 serum metabolites associated with milk-microbial score and 23 serum metabolites associated with yogurt-microbial score, respectively (FDR-corrected P < 0.1) (Fig 5a, b, Supplemental Table S3). Among the metabolites associated with milk-microbial score, we also observed a positive association between undecylenic acid and milk consumption (FDR-corrected P = 0.02), along with a negative association of 2-hydroxy-3-methylbutyric acid with milk consumption (FDR-corrected P = 0.006). Similarly, we found that 2-hydroxybutyric acid and L-alanine were negatively associated with yogurt consumption (both FDR-corrected P = 0.04) (Fig 5c). Further analysis revealed a correlation profile between serum metabolites and HDL cholesterol, triglycerides. Specifically, HDL cholesterol was positively associated with undecylenic acid, while negatively associated with L-alanine. Triglycerides was positively associated with 2-hydroxybutyric acid, L-alanine and 2-hydroxybutyric acid, while inversely associated with undecylenic acid (Fig 5b).

## 4 Discussion

In this prospective cohort study, we found that baseline habitual total dairy intake and yogurt intake were both positively associated with gut microbial diversity and with the abundance of specific genus. Those dairy-regulated gut microbial features were beneficially associated with cardiometabolic traits, such as blood triglycerides and HDL cholesterol. The metabolomics analysis showed that dairy-related gut microbiota was associated with the host circulating metabolomics profile, suggesting the beneficial association of dairy intake with the cardiometabolic risk factors.

Although some studies have explored the effects of dairy intakes on gut microbial diversity, their results were not consistent [6,[29], [30], [31]]. After a 3 week-daily ingestion of fermented milk products, the Shannon index of five healthy adults was decreased [29]. In contrast, several recent intervention studies did not find a significant effect of yogurt intake on gut microbial diversity [30,32]. For example, in a short-term (42 days) intervention of yogurt consumption, no difference for α-diversity was observed before and after the intervention [32]. On the population level, in a cross-sectional study from Netherlands (n = 1179), sour milk was positively associated with α-diversity, whereas drinking high-fat milk was inversely associated [6]. However, another cross-sectional study involving 862 participants showed that higher dairy product intake (including milk, cheese and other types) was correlated with lower α-diversity [5]. These inconsistencies may be due to the short-term and small sample size for the trial, or the cross-sectional nature for the conducted cohort study.

Accumulating evidence has shown the association between dairy intake and specific taxa [5,33]. In line with our results, Bifidobacterium—was increased in individuals with lactose malabsorption after four weeks whole milk intervention [34]. Habitual intake of milk or other dairy products (except cheese) was positively associated with Streptococcus in a cross-sectional analysis of 862 French adults [5], which was consistent with our current results. Nonetheless, some genus detected in our analysis have not been investigated in the context of their modulation by dairy intake yet, such as Lachnobacterium, Roseburia and Megasphaera. In the present study, unclassified genus of family Ruminococcaceae, unclassified genus of family Rikenellaceae and unclassified genus of family Barnesiellaceae were enriched in the highest yogurt intake group. Interestingly, all these dairy-related families were inversely associated with arterial stiffness reported in a previous study [17]. Moreover, as the biomarker of high dairy intake, several genera are producers of butyrate, such as Clostridium, Roseburia and Lachnobacterium. Butyrate is the major energy source of human colon cells and has a beneficial impact on glucose and energy homeostasis, as well as preventing gut microbiota dysbiosis [35].

We found that α-diversity indices (including Shannon index) and dairy-microbial scores were negatively associated with blood triglycerides. Shannon index represents the richness and evenness of the gut microbiota. Similarly, results from the LifeLines-DEEP cohort showed that after adjusting for age and sex, OTU richness was inversely correlated with blood triglycerides among 893 participants [36], and suggested that gut microbiota may contribute to the variation in blood lipids. Other cohort studies also supported that gut microbial diversity was associated with other cardiometabolic diseases, such as type 2 diabetes, hypertension and arterial stiffness [16,17,37]. For example, gut microbial diversity (Shannon index) was inversely associated arterial stiffness in 617 middle-age women from TwinsUK cohort [17]. Although gut microbial α-diversity seems to be a generally good indicator of a “healthy gut”, more mechanistic studies are required to clarify the causality [35]. In addition, although there are significant differences in β-diversity based on dairy consumption, there is so much overlap between dairy groups that β-diversity does not seems to be a reliable diagnostic, as reflected in R^2^.

Interestingly, results from our interaction analysis and subgroup analysis lead to a hypothesis that gut microbiome may mediate the association of dairy intake with blood lipids, although causality could not be proved in the present study. These novel results indicated that the beneficial effect of the gut microbiota diversity or dairy-associated microbial features on blood lipids may be abolished by a dietary background with low dairy intake. Our data suggested that L-alanine was inversely associated with yogurt-microbial score. Previous serum metabolomics study reported that this metabolite was positively"?> associated with incident cardiovascular disease among 3569 participants [38]. Their results also showed that alanine was positively associated with triglycerides while negatively associated with HDL cholesterol, which were consistent with our results. In addition, we found that both 2-hydroxy-3-methylbutyric acid and 2-hydroxybutyric acid were negatively associated with dairy consumption and dairy-microbial scores. It has been noted that elevated levels of 2-hydroxybutyric acid in the plasma is a good marker for early-stage type II diabetes [39]. Meanwhile, 2-hydroxy-3-methylbutyric acid is a metabolite found in the urine of patients with propionic acidemia and glyceroluria [40]. Taken together, these findings suggest that consumption of dairy foods, especially yogurt, may contribute to the prevention of cardiometabolic diseases [12,41,42].

The present study has several strengths. First, to the best of our knowledge, this is the first prospective cohort study to examine the association of dairy products with gut microbial diversity in a Chinese population. Second, we have a larger sample size compared with most available cross-sectional studies. Third, although the dairy intake (milk: mean 93.2 g/d; yogurt: mean 29.4 g/d) in our cohort is much lower than the recommendation of 300 g/d, it can reflect the national dairy consumption in China (mean 34.5 g/d) [34,43]. Few studies have investigated associations of dairy products with gut microbiota and cardiometabolic health with low overall dairy consumption. Despite the low level of intake, more dairy consumption is associated with cardiometabolic heath in previous study [44,45].

The study also has several limitations. First, this study is based on middle-aged and elderly Chinese with relatively low dairy intake, and may not be generalizable to other age groups or ethnicities. Second, our analysis only use information of dairy consumption at a single time-point at baseline, and dietary pattern may change over time. Nevertheless, we find that the dairy intake of the study participants does not change substantially during the follow-up duration (with fair ICC for each dairy variable). Third, our results were from an observation study and we cannot rule out the potential influence of some unmeasured confounders, more studies are needed to prove causality. Finally, all the participants are from the same city in China. Therefore, additional multi-centre and longitudinal studies are needed to confirm our results.

In conclusion, results from the present large Chinese cohort supports that total dairy intake and yogurt intake are prospectively associated with higher gut microbial diversity, and associated with gut microbial community structure. Those dairy-related gut microbial features are favourably associated with cardiometabolic risk factors. The present findings suggest that dairy consumption should not be discouraged and perhaps should even be encouraged, as to improve gut health and maintain cardiometabolic health in a population of low dairy intake. Nevertheless, more research is warranted to replicate our findings and to further investigate the role of gut microbiota in the link between diet and cardiometabolic health.

## Contributors

JSZ, YMC: designed research; LSYZ, CWL, FX: collected the data; MS, ZM, ZJ, YF: performed the data analysis; JSZ, MS: wrote the manuscript (MS drafted the initial manuscript; JSZ and MS finalized the manuscript); JSZ, YMC, WG, FX: critically reviewed the manuscript; JSZ and YMC: had primary responsibility for the final content; All authors read, revised and approved the final manuscript.

## Evidence before this study

Dairy products are important components of a common dietary pattern, which may play an important role for the prevention of cardiometabolic diseases. Growing evidence has revealed that the gut microbiota composition was associated with cardiometabolic diseases. However, only several moderate cross-sectional studies or short-term intervention trials indicated the effects of dairy consumption on the gut microbiota. In addition, little is known about the health benefit of increasing dairy intake for gut microbiota in a population with relatively low dairy intake.

## Added value of this study

This is a large longitudinal human cohort which suggests that higher dairy consumption is prospectively associated with a higher gut microbiota α-diversity. The dairy-related gut microbial features are favorably associated with cardiometabolic risk factors, such as blood triglycerides and HDL cholesterol. Dairy-related gut microbiota may influence the host circulating metabolomics profile, contributing to the beneficial association of dairy intake with the cardiometabolic risk factors.

## Implications of all the available evidence

Our findings suggest that dairy consumption should be encouraged for the prevention of cardiometabolic diseases in a general population with low dairy intake. The identified dairy intake-related gut microbiota and circulating metabolites biomarkers can serve as potential therapeutic targets for cardiometabolic diseases in future.

## Data sharing statement

The raw data of 16S rRNA sequencing is available in the CNSA (https://db.cngb.org/cnsa/) of CNGBdb at accession number CNP0000829. The metabolomics data was deposited at the Metabolomics Workbench (ST001669). Other datasets generated during and/or analyzed during the current study are available from the corresponding authors upon reasonable request.

## Declaration of Competing Interest

The authors declare no conflict of interest.

## Appendix Supplementary materials

Image, application 1
