# Supplementary material for: Multi-omics analyses reveal relationships among dairy consumption, gut microbiota and cardiometabolic health
Source: eBioMedicine. 2021 Mar 19;66:103284. doi: 10.1016/j.ebiom.2021.103284 (PMC7985282; doi:10.1016/j.ebiom.2021.103284)
Supplement: Supplementary file 1 [file mmc1.docx]

**Multi-omics analyses reveal relationships among dairy consumption, gut microbiota and cardiometabolic health**

**Supplementary Material**

**Contents**

**Table S1.** Association of dairy intake with the dairy-microbial score generated from 16S data

**Table S2.** Associations between dairy-related gut microbial features and cardiometabolic risk factors (16S data)

**Table S3.** The concentrations of metabolites by groups of total dairy consumption (umol/L).

**Table S4.** The raw reads of 16S rRNA sequencing in our study (n = 1780).

**Fig S1.** Flow diagram of participants’ selection for the analyses of present study

**Fig S2.** Forest plot for the interaction of dairy consumption with gut microbial features on blood triglycerides.

**Table S1.** Association of dairy intake with the dairy microbial score generated from 16S data^a^

| **Dataset** | **Variable** | **Beta (95% CI)** | ***P*** |
| --- | --- | --- | --- |
| **16S (n = 1780)** | Total dairy-microbial score^b^ |  |  |
|  | Model 1 | 0.19 (0.05, 0.33) | 0.009 |
|  | Model 2 | 0.20 (0.05, 0.35) | 0.007 |
|  | Model 3 | 0.21 (0.06, 0.36) | 0.006 |
|  | Milk-microbial score |  |  |
|  | Model 1 | 0.23 (0.09, 0.37) | 0.001 |
|  | Model 2 | 0.24 (0.10, 0.38) | <0.001 |
|  | Model 3 | 0.24 (0.10, 0.39) | <0.001 |
|  | Yogurt-microbial score |  |  |
|  | Model 1 | 0.80 (0.40, 1.19) | <0.001 |
|  | Model 2 | 0.80 (0.40, 1.19) | <0.001 |
|  | Model 3 | 0.74 (0.34, 1.15) | <0.001 |

^a^Beta (95% CI) and *P* were calculated in a linear regression model to examine the association of dairy intake with corresponding dairy-microbial score, after multivariable adjustment, with following models: model 1, age and sex; model 2, further adjusted by BMI, total energy intake, physical activity, smoking status, drinking status, education attainment and household income level; and model 3, further adjusted for dietary intakes, including vegetable, fruit, fish, egg and red meat. All models were estimated for each corresponding dairy variable.

^b^The dairy microbial scores were transformed to z score.

**Table S2.** Associations between dairy-related gut microbial features and cardiometabolic risk factors^a^ (16S data)

| **Cardiometabolic traits (SD)** | **Gut microbial features** | **N** | **Beta (95% CI)** | ***P*** | **FDR**^c^ |
| --- | --- | --- | --- | --- | --- |
| **TC (1.08 mmol/l)** | Dairy-microbial scores |  |  |  |  |
|  | Total dairy-microbial score | 1713 | 0.03 (-0.02, 0.07) | 0.25 | 0.59 |
|  | Milk-microbial score | 1713 | 0.02 (-0.02, 0.07) | 0.31 | 0.68 |
|  | Yogurt-microbial score | 1713 | -0.03 (-0.08, 0.02) | 0.20 | 0.52 |
|  | Alpha-diversity indices |  |  |  |  |
|  | Shannon | 1713 | 0.02 (-0.03, 0.07) | 0.37 | 0.68 |
|  | Simpson | 1713 | 0.002 (-0.05, 0.05) | 0.93 | 0.96 |
|  | Observed species | 1713 | 0.04 (-0.02, 0.09) | 0.17 | 0.45 |
| **HDL-C (log-transformed, 0.26)** ^b^ | Dairy-microbial scores |  |  |  |  |
|  | Total dairy-microbial score | 1713 | 0.06 (0.02, 0.11) | 0.005 | 0.038 |
|  | Milk-microbial score | 1713 | 0.06 (0.02, 0.11) | 0.004 | 0.033 |
|  | Yogurt-microbial score | 1713 | -0.01 (-0.06, 0.03) | 0.54 | 0.79 |
|  | Alpha-diversity indices |  |  |  |  |
|  | Shannon | 1713 | 0.07 (0.02, 0.12) | 0.003 | 0.029 |
|  | Simpson | 1713 | 0.05 (0.004, 0.1) | 0.032 | 0.17 |
|  | Observed species | 1713 | 0.06 (0.01, 0.1) | 0.023 | 0.14 |
| **LDL-C (0.98 mmol/l)** | Dairy-microbial scores |  |  |  |  |
|  | Total dairy-microbial score | 1713 | 0.01 (-0.04, 0.05) | 0.81 | 0.96 |
|  | Milk-microbial score | 1713 | 0.003 (-0.04, 0.05) | 0.91 | 0.96 |
|  | Yogurt-microbial score | 1713 | 0.005 (-0.04, 0.05) | 0.85 | 0.96 |
|  | Alpha-diversity indices |  |  |  |  |
|  | Shannon | 1713 | 0.04 (-0.01, 0.09) | 0.11 | 0.35 |
|  | Simpson | 1713 | 0.01 (-0.04, 0.06) | 0.77 | 0.96 |
|  | Observed species | 1713 | 0.07 (0.02, 0.12) | 0.01 | 0.06 |
| **TC/HDL-C (log-transformed, 0.28)** ^b^ | Dairy-microbial scores |  |  |  |  |
|  | Total dairy-microbial score | 1713 | -0.04 (-0.09, 0.01) | 0.09 | 0.30 |
|  | Milk-microbial score | 1713 | -0.04 (-0.09, 0.002) | 0.06 | 0.25 |
|  | Yogurt-microbial score | 1713 | -0.01 (-0.06, 0.03) | 0.61 | 0.86 |
|  | Alpha-diversity indices |  |  |  |  |
|  | Shannon | 1713 | -0.05 (-0.1, 0.003) | 0.06 | 0.25 |
|  | Simpson | 1713 | -0.04 (-0.09, 0.005) | 0.08 | 0.27 |
|  | Observed species | 1713 | -0.02 (-0.08, 0.03) | 0.37 | 0.68 |
| **TG (log-transformed, 0.50)** ^b^ | Dairy-microbial scores |  |  |  |  |
|  | Total dairy-microbial score | 1713 | -0.08 (-0.12, -0.03) | 0.002 | 0.018 |
|  | Milk-microbial score | 1713 | -0.08 (-0.13, -0.03) | <0.001 | 0.013 |
|  | Yogurt-microbial score | 1713 | -0.08 (-0.13, -0.04) | <0.001 | 0.012 |
|  | Alpha-diversity indices |  |  |  |  |
|  | Shannon | 1713 | -0.13 (-0.18, -0.08) | <0.001 | <0.001 |
|  | Simpson | 1713 | -0.08 (-0.13, -0.03) | <0.001 | 0.013 |
|  | Observed species | 1713 | -0.14 (-0.19, -0.09) | <0.001 | <0.001 |
| **SBP (16.9 mmHg)** | Dairy-microbial scores |  |  |  |  |
|  | Total dairy-microbial score | 1767 | -0.004 (-0.05, 0.04) | 0.87 | 0.96 |
|  | Milk-microbial score | 1767 | -0.005 (-0.05, 0.04) | 0.83 | 0.96 |
|  | Yogurt-microbial score | 1767 | -0.01 (-0.05, 0.04) | 0.81 | 0.96 |
|  | Alpha-diversity indices |  |  |  |  |
|  | Shannon | 1767 | -0.001 (-0.05, 0.05) | 0.98 | 0.99 |
|  | Simpson | 1767 | -0.01 (-0.06, 0.04) | 0.74 | 0.96 |
|  | Observed species | 1767 | 0.01 (-0.04, 0.06) | 0.76 | 0.96 |
| **DBP (12.3 mmHg)** | Dairy-microbial scores |  |  |  |  |
|  | Total dairy-microbial score | 1767 | -0.02 (-0.07, 0.03) | 0.40 | 0.70 |
|  | Milk-microbial score | 1767 | -0.02 (-0.06, 0.03) | 0.43 | 0.72 |
|  | Yogurt-microbial score | 1767 | 0.01 (-0.04, 0.06) | 0.67 | 0.90 |
|  | Alpha-diversity indices |  |  |  |  |
|  | Shannon | 1767 | 0.04 (-0.01, 0.08) | 0.16 | 0.45 |
|  | Simpson | 1767 | 0.01 (-0.04, 0.06) | 0.64 | 0.88 |
|  | Observed species | 1767 | 0.05 (-0.004, 0.1) | 0.07 | 0.26 |
| **BMI (3.23 kg/m^2^)** | Dairy-microbial scores |  |  |  |  |
|  | Total dairy-microbial score | 1767 | -0.02 (-0.21, 0.09) | 0.45 | 0.72 |
|  | Milk-microbial score | 1767 | -0.02 (-0.21, 0.09) | 0.45 | 0.72 |
|  | Yogurt-microbial score | 1767 | -0.002 (-0.16, 0.15) | 0.93 | 0.96 |
|  | Alpha-diversity indices |  |  |  |  |
|  | Shannon | 1767 | -0.03 (-0.26, 0.07) | 0.25 | 0.59 |
|  | Simpson | 1767 | -0.04 (-0.27, 0.05) | 0.16 | 0.45 |
|  | Observed species | 1767 | 0.002 (-0.16, 0.18) | 0.93 | 0.96 |
| **Waist circumference (8.98 cm)** | Dairy-microbial scores |  |  |  |  |
|  | Total dairy-microbial score | 1754 | -0.03 (-0.06, -0.001) | 0.040 | 0.19 |
|  | Milk-microbial score | 1754 | -0.03 (-0.06, -0.003) | 0.033 | 0.17 |
|  | Yogurt-microbial score | 1754 | -0.01 (-0.04, 0.02) | 0.47 | 0.72 |
|  | Alpha-diversity indices |  |  |  |  |
|  | Shannon | 1754 | -0.02 (-0.05, 0.01) | 0.12 | 0.36 |
|  | Simpson | 1754 | -0.03 (-0.06, 0.001) | 0.06 | 0.25 |
|  | Observed species | 1754 | -0.01 (-0.04, 0.02) | 0.51 | 0.76 |
| **Hba1c (0.54 %)** | Dairy-microbial scores |  |  |  |  |
|  | Total dairy-microbial score | 1090 | 0.003 (-0.05, 0.06) | 0.93 | 0.96 |
|  | Milk-microbial score | 1090 | -0.0001 (-0.06, 0.06) | 0.99 | 0.99 |
|  | Yogurt-microbial score | 1090 | -0.01 (-0.06, 0.05) | 0.83 | 0.96 |
|  | Alpha-diversity indices |  |  |  |  |
|  | Shannon | 1090 | -0.03 (-0.1, 0.03) | 0.34 | 0.68 |
|  | Simpson | 1090 | -0.01 (-0.07, 0.05) | 0.81 | 0.96 |
|  | Observed species | 1090 | -0.02 (-0.09, 0.04) | 0.47 | 0.72 |
| **Glucose (log-transformed, 0.15)** ^b^ | Dairy-microbial scores |  |  |  |  |
|  | Total dairy-microbial score | 1712 | -0.02 (-0.07, 0.03) | 0.37 | 0.68 |
|  | Milk-microbial score | 1712 | -0.02 (-0.07, 0.02) | 0.33 | 0.68 |
|  | Yogurt-microbial score | 1712 | 0.01 (-0.03, 0.06) | 0.57 | 0.82 |
|  | Alpha-diversity indices |  |  |  |  |
|  | Shannon | 1712 | -0.02 (-0.07, 0.03) | 0.43 | 0.72 |
|  | Simpson | 1712 | -0.03 (-0.07, 0.02) | 0.29 | 0.66 |
|  | Observed species | 1712 | 0.02 (-0.03, 0.08) | 0.35 | 0.68 |

^a^Associations were expressed as the difference in cardiometabolic risk factors (in SD units) per 1 SD difference in each gut microbial feature. Beta (95% CI) and *P* were calculated in a linear regression model after adjustment for age, sex, BMI, smoking status, drinking status, education attainment, household income level, total energy intake, physical activity, dietary intakes of vegetables, fruit, fish, egg and red meat. Alpha-diversity indices were also adjusted by technique confounders, such sequencing depth and sequencing run. TC, total cholesterol. HDL-C, high density lipoprotein cholesterol. LDL-C, low density lipoprotein cholesterol. TG, triglycerides. SBP, systolic blood pressure. DBP, diastolic blood pressure. BMI, body mass index. HbA1c, hemoglobin A1c. Glucose, Fasting blood glucose.

^b^Values were log-transformed.

^c^FDR-corrected *P* values.

**Table S3.** The concentrations of metabolites by groups of total dairy consumption (umol/L)^a^.

|  |  |  | **Group 1** | **Group 2** | **Group 3** | **Group 4** | **Total** |
| --- | --- | --- | --- | --- | --- | --- | --- |
| Groups, servings |  |  | <1/mo | 1/mo-1/wk | 1/wk-0.5/d | ≥0.5/d |  |
| Participants |  |  | 109 | 128 | 295 | 416 | 948 |
| HMDB0003152 | N_Methylnicotinamide | Pyridines | 14.1 (11.8) | 14.4 (14.2) | 14.8 (15.1) | 14.0 (12.2) | 14.3 (13.4) |
| HMDB0011743 | 2_Phenylpropionate | Phenylpropanoic acids | 1.0 (1.7) | 1.3 (2.8) | 0.9 (1.6) | 1.0 (2.2) | 1.0 (2.1) |
| HMDB0000764 | Hydrocinnamic acid | Phenylpropanoic acids | 1.0 (1.9) | 1.4 (2.9) | 0.9 (1.7) | 1.0 (2.3) | 1.0 (2.2) |
| HMDB0000208 | Oxoglutaric acid | Organic acids | 17.7 (8.2) | 17.1 (5.8) | 16.0 (5.5) | 16.6 (6.7) | 16.6 (6.5) |
| HMDB0000008 | 2_Hydroxybutyric acid | Organic acids | 77.3 (26.3) | 80.7 (38.5) | 74.6 (23.2) | 74.6 (25.0) | 75.7 (26.9) |
| HMDB0000661 | Glutaric acid | Organic acids | 0.8 (0.9) | 0.6 (0.5) | 0.7 (0.5) | 0.7 (0.6) | 0.7 (0.6) |
| HMDB0002302 | 3_Indolepropionic acid | Indoles | 1.8 (6.6) | 2.3 (7.1) | 1.4 (4.8) | 2.9 (9.0) | 2.2 (7.4) |
| HMDB0000197 | Indoleacetic acid | Indoles | 3.2 (2.3) | 3.0 (2.1) | 2.9 (1.9) | 2.9 (2.0) | 3.0 (2.0) |
| HMDB0000407 | 2_Hydroxy_3_methylbutyric acid | Fatty acids | 17.9 (10.9) | 17.0 (12.5) | 13.8 (5.1) | 13.3 (5.6) | 14.5 (7.7) |
| HMDB0033724 | Undecylenic acid | Fatty acids | 14.7 (7.6) | 15.6 (10.4) | 16.9 (10.1) | 18.4 (11.5) | 17.2 (10.6) |
| HMDB0000039 | Butyric acid | Fatty acids | 1.1 (0.8) | 1.1 (0.6) | 1.0 (0.8) | 1.0 (0.6) | 1.1 (0.7) |
| HMDB0001844 | Methylsuccinic acid | Fatty acids | 0.3 (0.4) | 0.2 (0.2) | 0.3 (0.2) | 0.3 (0.2) | 0.3 (0.2) |
| HMDB0000222 | Palmitoylcarnitine | Carnitines | 0.3 (0.1) | 0.3 (0.1) | 0.3 (0.1) | 0.3 (0.1) | 0.3 (0.1) |
| HMDB0000619 | Cholic acid | Bile acids | 0.2 (0.4) | 0.2 (0.3) | 0.2 (0.4) | 0.2 (0.4) | 0.2 (0.4) |
| HMDB0000626 | Deoxycholic acid | Bile acids | 0.6 (0.7) | 0.7 (0.7) | 0.6 (0.8) | 0.6 (0.8) | 0.6 (0.8) |
| HMDB0002639 | GLCA_3S | Bile acids | 0.3 (0.3) | 0.3 (0.3) | 0.3 (0.3) | 0.3 (0.3) | 0.3 (0.3) |
| HMDB0000631 | Glycodeoxycholic acid | Bile acids | 0.4 (0.6) | 0.5 (1.4) | 0.6 (1.4) | 0.4 (1.2) | 0.5 (1.3) |
| HMDB0000708 | Glycoursodeoxycholic acid | Bile acids | 0.5 (0.8) | 0.3 (0.4) | 0.4 (0.6) | 0.4 (0.6) | 0.4 (0.6) |
| HMDB0000733 | Hyodeoxycholic acid | Bile acids | 0.1 (0.1) | 0.1 (0.2) | 0.1 (0.1) | 0.1 (0.1) | 0.1 (0.1) |
| HMDB0000714 | Hippuric acid | Benzenoids | 3.9 (4.3) | 4.1 (4.5) | 4.3 (4.9) | 3.9 (4.1) | 4.1 (4.5) |
| HMDB0000209 | Phenylacetic acid | Benzenoids | 16.1 (13.8) | 15.8 (15.1) | 17.0 (17.2) | 15.9 (13.7) | 16.2 (15.1) |
| HMDB0000779 | Phenyllactic acid | Benzenoids | 0.4 (0.2) | 0.4 (0.2) | 0.3 (0.1) | 0.3 (0.2) | 0.3 (0.2) |
| HMDB0000167 | L_threonine | Amino acids | 123.7 (31.9) | 120.4 (27.4) | 118.7 (24.4) | 117.9 (25.7) | 119.2 (26.3) |
| HMDB0006344 | Alpha_N_Phenylacetyl_L_glutamine | Amino acids | 2.1 (1.6) | 2.1 (1.9) | 2.1 (1.6) | 2.1 (1.9) | 2.1 (1.8) |
| HMDB0000064 | Creatine | Amino acids | 43.3 (15.2) | 44.7 (16.0) | 47.5 (17.4) | 45.3 (16.8) | 45.7 (16.7) |
| HMDB0000161 | L_Alanine | Amino acids | 431.3 (100.2) | 414.0 (107.9) | 401.2 (91.3) | 398.7 (96.3) | 405.3 (97.3) |
| HMDB0000148 | L_Glutamic acid | Amino acids | 22.3 (6.2) | 21.7 (6.0) | 21.3 (5.7) | 21.1 (5.9) | 21.4 (5.9) |

^a^Data are mean (SD).

**Table S4.** The raw reads of 16S rRNA sequencing in our study (n = 1780).

| ID | Raw_reads_number | q20 | q30 | ID | Raw_reads_number | q20 | q30 |
| --- | --- | --- | --- | --- | --- | --- | --- |
| D0001 | 50477 | 99.292 | 96.812 | D0891 | 41705 | 99.352 | 97.048 |
| D0002 | 31454 | 98.296 | 97.371 | D0892 | 38159 | 98.09 | 97.073 |
| D0003 | 41002 | 98.008 | 97.048 | D0893 | 51761 | 98.438 | 97.656 |
| D0004 | 31918 | 98.181 | 97.307 | D0894 | 41197 | 98.172 | 97.157 |
| D0005 | 28678 | 98.644 | 97.943 | D0895 | 40471 | 99.354 | 97.086 |
| D0006 | 37591 | 98.599 | 97.836 | D0896 | 37335 | 99.221 | 96.59 |
| D0007 | 56574 | 99.355 | 97.039 | D0897 | 53404 | 98.872 | 97.152 |
| D0008 | 58473 | 99.399 | 97.232 | D0898 | 38485 | 99.389 | 97.187 |
| D0009 | 27764 | 97.454 | 96.101 | D0899 | 33110 | 98.238 | 97.33 |
| D0010 | 37105 | 98.016 | 96.906 | D0900 | 46437 | 97.979 | 97.062 |
| D0011 | 31239 | 99.36 | 96.954 | D0901 | 50262 | 98.617 | 97.939 |
| D0012 | 37118 | 98.489 | 97.708 | D0902 | 53785 | 98.387 | 97.545 |
| D0013 | 36652 | 99.384 | 97.071 | D0903 | 57923 | 98.438 | 97.739 |
| D0014 | 51341 | 97.934 | 96.958 | D0904 | 41251 | 99.402 | 97.227 |
| D0015 | 39476 | 98.589 | 97.88 | D0905 | 54849 | 98.614 | 97.937 |
| D0016 | 37041 | 99.413 | 97.225 | D0906 | 41212 | 98.474 | 97.711 |
| D0017 | 39081 | 99.326 | 96.803 | D0907 | 33510 | 99.358 | 97.051 |
| D0018 | 38580 | 98.12 | 97.233 | D0908 | 31852 | 98.148 | 97.034 |
| D0019 | 41596 | 98.549 | 97.825 | D0909 | 42994 | 99.063 | 96.136 |
| D0020 | 36218 | 98.092 | 97.099 | D0910 | 48677 | 99.206 | 96.512 |
| D0021 | 36419 | 96.764 | 95.302 | D0911 | 35950 | 99.392 | 97.216 |
| D0022 | 48040 | 98.305 | 97.459 | D0912 | 31652 | 99.223 | 96.597 |
| D0023 | 43222 | 98.564 | 97.787 | D0913 | 29012 | 98.646 | 97.921 |
| D0024 | 46227 | 98.457 | 97.75 | D0914 | 34824 | 99.358 | 97.096 |
| D0025 | 34311 | 98.189 | 97.381 | D0915 | 29089 | 94.116 | 91.682 |
| D0026 | 35070 | 99.369 | 96.998 | D0916 | 34647 | 97.583 | 96.532 |
| D0027 | 67058 | 99.373 | 97.012 | D0917 | 39240 | 96.444 | 94.81 |
| D0028 | 47669 | 98.481 | 97.747 | D0918 | 36756 | 99.315 | 96.966 |
| D0029 | 26789 | 99.407 | 97.148 | D0919 | 35351 | 98.217 | 97.381 |
| D0030 | 43449 | 98.54 | 97.77 | D0920 | 49246 | 98.133 | 97.3 |
| D0031 | 48662 | 98.283 | 97.512 | D0921 | 40538 | 98.025 | 97.192 |
| D0032 | 41838 | 99.338 | 96.893 | D0922 | 16239 | 97.201 | 96.013 |
| D0033 | 47044 | 98.519 | 97.786 | D0923 | 36539 | 99.322 | 96.98 |
| D0034 | 44495 | 99.363 | 96.957 | D0924 | 52172 | 98.542 | 97.794 |
| D0035 | 44962 | 99.185 | 96.377 | D0925 | 30423 | 98.113 | 97.277 |
| D0036 | 44328 | 98.619 | 97.937 | D0926 | 58935 | 98.772 | 98.111 |
| D0037 | 45593 | 99.41 | 97.179 | D0927 | 38842 | 97.54 | 96.385 |
| D0038 | 50183 | 99.294 | 96.82 | D0928 | 55065 | 99.185 | 97.54 |
| D0039 | 49065 | 98.313 | 97.458 | D0929 | 48973 | 98.519 | 97.768 |
| D0040 | 37828 | 98.163 | 97.355 | D0930 | 39985 | 99.341 | 97.016 |
| D0041 | 33203 | 99.345 | 96.941 | D0931 | 51753 | 98.618 | 97.882 |
| D0042 | 36789 | 98.587 | 97.851 | D0932 | 40815 | 99.41 | 97.277 |
| D0043 | 53063 | 99.371 | 97.01 | D0933 | 44948 | 97.7 | 96.642 |
| D0044 | 46522 | 99.392 | 97.127 | D0934 | 36188 | 98.345 | 97.605 |
| D0045 | 30209 | 99.321 | 96.983 | D0935 | 39492 | 98.412 | 97.613 |
| D0046 | 37091 | 99.412 | 97.196 | D0936 | 33910 | 99.139 | 96.367 |
| D0047 | 59107 | 98.429 | 96.321 | D0937 | 53597 | 98.422 | 97.661 |
| D0048 | 46099 | 99.28 | 96.692 | D0938 | 34459 | 97.951 | 96.979 |
| D0049 | 27525 | 99.241 | 96.57 | D0939 | 39370 | 99.397 | 97.246 |
| D0050 | 37309 | 98.307 | 97.486 | D0940 | 43864 | 98.325 | 97.585 |
| D0051 | 29049 | 99.363 | 96.984 | D0941 | 42143 | 98.576 | 97.902 |
| D0052 | 40016 | 98.621 | 97.927 | D0942 | 66338 | 99.364 | 96.974 |
| D0053 | 47486 | 99.387 | 97.199 | D0943 | 50867 | 99.307 | 96.855 |
| D0054 | 44103 | 98.567 | 97.792 | D0944 | 37004 | 98.469 | 97.589 |
| D0055 | 27661 | 94.727 | 92.263 | D0945 | 53220 | 99.378 | 97.011 |
| D0056 | 28362 | 99.254 | 96.587 | D0946 | 41573 | 98.55 | 97.89 |
| D0057 | 39538 | 98.609 | 97.929 | D0947 | 55977 | 98.561 | 97.87 |
| D0058 | 32897 | 97.258 | 95.943 | D0948 | 50113 | 97.571 | 96.498 |
| D0059 | 38751 | 95.566 | 93.68 | D0949 | 39158 | 99.393 | 97.21 |
| D0060 | 45220 | 98.351 | 97.513 | D0950 | 41631 | 95.071 | 92.896 |
| D0061 | 35463 | 99.426 | 97.259 | D0951 | 38478 | 99.398 | 97.241 |
| D0062 | 39857 | 98.681 | 98.041 | D0952 | 30207 | 99.377 | 97.16 |
| D0063 | 46452 | 99.39 | 97.109 | D0953 | 40135 | 99.391 | 97.223 |
| D0064 | 41956 | 97.451 | 96.273 | D0954 | 31790 | 99.339 | 97.059 |
| D0065 | 34084 | 98.44 | 97.572 | D0955 | 47055 | 99.385 | 97.208 |
| D0066 | 46913 | 98.643 | 97.978 | D0956 | 36449 | 99.197 | 96.618 |
| D0067 | 46899 | 98.21 | 97.287 | D0957 | 40923 | 99.409 | 97.271 |
| D0068 | 36780 | 98.24 | 97.308 | D0958 | 36673 | 99.361 | 97.098 |
| D0069 | 34118 | 98.4 | 97.496 | D0959 | 35222 | 99.399 | 97.224 |
| D0070 | 50080 | 99.368 | 97.014 | D0960 | 32577 | 99.313 | 96.919 |
| D0071 | 42492 | 97.832 | 96.869 | D0961 | 32627 | 99.384 | 97.17 |
| D0072 | 38047 | 97.425 | 96.172 | D0962 | 48523 | 98.476 | 97.613 |
| D0073 | 37323 | 97.068 | 95.688 | D0963 | 36070 | 98.214 | 97.393 |
| D0074 | 28471 | 99.423 | 97.248 | D0964 | 42214 | 98.664 | 97.901 |
| D0075 | 42521 | 98.036 | 96.947 | D0965 | 46146 | 97.783 | 96.825 |
| D0076 | 60763 | 99.285 | 96.784 | D0966 | 48458 | 98.556 | 97.888 |
| D0077 | 33362 | 98.623 | 97.892 | D0967 | 31679 | 99.352 | 96.938 |
| D0078 | 42003 | 98.348 | 97.431 | D0968 | 37572 | 99.391 | 97.106 |
| D0079 | 29243 | 98.637 | 97.901 | D0969 | 45491 | 98.056 | 97.17 |
| D0080 | 46343 | 98.683 | 97.984 | D0970 | 49020 | 98.534 | 97.775 |
| D0081 | 41760 | 98.197 | 97.322 | D0971 | 37865 | 99.401 | 97.252 |
| D0082 | 35648 | 97.524 | 96.286 | D0972 | 47585 | 98.498 | 97.754 |
| D0083 | 65501 | 99.343 | 96.983 | D0973 | 40537 | 98.585 | 97.811 |
| D0084 | 57029 | 99.416 | 97.187 | D0974 | 38636 | 98.264 | 97.417 |
| D0085 | 32391 | 98.448 | 97.715 | D0975 | 31790 | 97.486 | 96.341 |
| D0086 | 39677 | 98.468 | 97.666 | D0976 | 50426 | 98.457 | 97.646 |
| D0087 | 42704 | 98.599 | 97.886 | D0977 | 53093 | 98.155 | 97.268 |
| D0088 | 66054 | 98.52 | 97.767 | D0978 | 35381 | 98.426 | 97.609 |
| D0089 | 40982 | 97.788 | 96.779 | D0979 | 45634 | 98.493 | 97.793 |
| D0090 | 58056 | 98.378 | 97.568 | D0980 | 45547 | 98.119 | 97.12 |
| D0091 | 46054 | 98.592 | 97.786 | D0981 | 39527 | 99.343 | 97.003 |
| D0092 | 38786 | 98.451 | 97.69 | D0982 | 50258 | 98.547 | 97.842 |
| D0093 | 36519 | 97.668 | 96.621 | D0983 | 41603 | 98.589 | 97.848 |
| D0094 | 45544 | 98.003 | 97.036 | D0984 | 45687 | 98.247 | 97.401 |
| D0095 | 44527 | 97.481 | 96.417 | D0985 | 43562 | 98.612 | 97.919 |
| D0096 | 51497 | 98.162 | 97.24 | D0986 | 34254 | 99.363 | 97.092 |
| D0097 | 42988 | 98.157 | 97.257 | D0987 | 33233 | 97.472 | 96.302 |
| D0098 | 54610 | 98.055 | 97.115 | D0988 | 53702 | 98.251 | 97.461 |
| D0099 | 37884 | 98.584 | 97.855 | D0989 | 49426 | 97.318 | 96.188 |
| D0100 | 42481 | 98.059 | 96.978 | D0990 | 78539 | 98.502 | 97.836 |
| D0101 | 47625 | 98.527 | 97.548 | D0991 | 44260 | 98.659 | 97.963 |
| D0102 | 43325 | 98.332 | 97.54 | D0992 | 41229 | 99.297 | 96.671 |
| D0103 | 37416 | 96.862 | 95.223 | D0993 | 40295 | 99.283 | 96.825 |
| D0104 | 39666 | 98.661 | 97.937 | D0994 | 41388 | 98.555 | 97.826 |
| D0105 | 62151 | 99.138 | 96.123 | D0995 | 41019 | 96.742 | 95.303 |
| D0106 | 30544 | 98.338 | 97.497 | D0996 | 31961 | 98.051 | 97.191 |
| D0107 | 68632 | 98.019 | 96.826 | D0997 | 57347 | 98.285 | 97.497 |
| D0108 | 31205 | 97.294 | 96.11 | D0998 | 43138 | 98.371 | 97.544 |
| D0109 | 27495 | 98.391 | 97.562 | D0999 | 41673 | 97.634 | 96.544 |
| D0110 | 23233 | 98.386 | 97.565 | D1000 | 37474 | 99.358 | 97.068 |
| D0111 | 36089 | 98.298 | 97.399 | D1001 | 42530 | 98.634 | 97.9 |
| D0112 | 47229 | 99.261 | 96.774 | D1002 | 44263 | 98.623 | 97.839 |
| D0113 | 33541 | 98.669 | 97.965 | D1003 | 37346 | 99.37 | 96.999 |
| D0114 | 37708 | 97.974 | 97.006 | D1004 | 59379 | 99.138 | 96.231 |
| D0115 | 35613 | 98.154 | 97.288 | D1005 | 42791 | 99.296 | 96.904 |
| D0116 | 37782 | 99.311 | 96.775 | D1006 | 50871 | 94.525 | 92.28 |
| D0117 | 27853 | 99.361 | 96.989 | D1007 | 49671 | 98.67 | 98.011 |
| D0118 | 33810 | 98.67 | 97.941 | D1008 | 30194 | 99.367 | 97.034 |
| D0119 | 39961 | 99.199 | 96.444 | D1009 | 81776 | 99.386 | 97.078 |
| D0120 | 60652 | 99.139 | 97.502 | D1010 | 78231 | 99.411 | 97.212 |
| D0121 | 30752 | 98.267 | 97.343 | D1011 | 42643 | 98.439 | 97.698 |
| D0122 | 41823 | 98.587 | 97.774 | D1012 | 35902 | 99.339 | 97 |
| D0123 | 36489 | 98.096 | 97.164 | D1013 | 33334 | 98.011 | 97.134 |
| D0124 | 26601 | 96.829 | 95.366 | D1014 | 43608 | 97.818 | 96.776 |
| D0125 | 36665 | 98.499 | 97.731 | D1015 | 33690 | 98.19 | 97.273 |
| D0126 | 35917 | 98.27 | 97.508 | D1016 | 37622 | 98.313 | 97.546 |
| D0127 | 27803 | 98.578 | 97.84 | D1017 | 36549 | 99.386 | 97.19 |
| D0128 | 43987 | 98.566 | 97.869 | D1018 | 35801 | 98.032 | 97.223 |
| D0129 | 47211 | 98.442 | 97.679 | D1019 | 46265 | 98.552 | 97.832 |
| D0130 | 44884 | 98.68 | 98.055 | D1020 | 25807 | 98.251 | 97.45 |
| D0131 | 45463 | 98.579 | 97.874 | D1021 | 87476 | 98.054 | 97.146 |
| D0132 | 21416 | 96.893 | 95.534 | D1022 | 39991 | 98.587 | 97.903 |
| D0133 | 41224 | 97.608 | 96.567 | D1023 | 36276 | 97.736 | 96.641 |
| D0134 | 42498 | 97.177 | 95.86 | D1024 | 42565 | 99.183 | 96.555 |
| D0135 | 16649 | 97.691 | 96.437 | D1025 | 32799 | 99.318 | 96.948 |
| D0136 | 39837 | 98.198 | 97.254 | D1026 | 33203 | 99.331 | 97.031 |
| D0137 | 35658 | 96.93 | 95.432 | D1027 | 41246 | 98.466 | 97.706 |
| D0138 | 40266 | 98.341 | 97.518 | D1028 | 26548 | 99.373 | 97.157 |
| D0139 | 27834 | 98.626 | 97.792 | D1029 | 48663 | 99.372 | 97.127 |
| D0140 | 40984 | 99.3 | 96.911 | D1030 | 49915 | 98.633 | 97.907 |
| D0141 | 50412 | 98.579 | 97.765 | D1031 | 43664 | 98.047 | 97.133 |
| D0142 | 30703 | 98.533 | 97.753 | D1032 | 40514 | 99.43 | 97.359 |
| D0143 | 54282 | 99.142 | 96.279 | D1033 | 47620 | 98.592 | 97.864 |
| D0144 | 31285 | 98.441 | 97.652 | D1034 | 45376 | 98.641 | 97.852 |
| D0145 | 34417 | 99.316 | 96.831 | D1035 | 39733 | 97.999 | 96.962 |
| D0146 | 40650 | 97.916 | 96.815 | D1036 | 43758 | 98.554 | 97.729 |
| D0147 | 37494 | 99.365 | 97.096 | D1037 | 45702 | 99.388 | 97.194 |
| D0148 | 34949 | 98.312 | 97.379 | D1038 | 40572 | 98.497 | 97.769 |
| D0149 | 36452 | 98.118 | 97.03 | D1039 | 41787 | 98.36 | 97.59 |
| D0150 | 34363 | 98.109 | 97.254 | D1040 | 46611 | 98.389 | 97.689 |
| D0151 | 142958 | 95.148 | 92.901 | D1041 | 40181 | 98.528 | 97.704 |
| D0152 | 33611 | 97.892 | 96.98 | D1042 | 41372 | 98.617 | 97.919 |
| D0153 | 40746 | 98.574 | 97.865 | D1043 | 31138 | 95.833 | 94.044 |
| D0154 | 41978 | 97.775 | 96.261 | D1044 | 33633 | 99.375 | 97.172 |
| D0155 | 25070 | 98.23 | 97.315 | D1045 | 48670 | 98.504 | 97.74 |
| D0156 | 27718 | 98.583 | 97.82 | D1046 | 34400 | 95.341 | 93.212 |
| D0157 | 38163 | 97.518 | 96.372 | D1047 | 37049 | 98.173 | 97.283 |
| D0158 | 46440 | 98.131 | 97.239 | D1048 | 40112 | 99.308 | 96.942 |
| D0159 | 29702 | 95.799 | 93.961 | D1049 | 39353 | 99.359 | 97.096 |
| D0160 | 24234 | 97.879 | 96.917 | D1050 | 36532 | 98.635 | 97.97 |
| D0161 | 44490 | 98.324 | 97.443 | D1051 | 43468 | 99.31 | 96.869 |
| D0162 | 41662 | 98.283 | 97.416 | D1052 | 36477 | 99.419 | 97.323 |
| D0163 | 40565 | 96.89 | 95.495 | D1053 | 58013 | 98.61 | 96.73 |
| D0164 | 34226 | 98.671 | 97.983 | D1054 | 65921 | 98.383 | 97.624 |
| D0165 | 30630 | 98.508 | 97.761 | D1055 | 71138 | 98.551 | 97.796 |
| D0166 | 41720 | 98.215 | 97.445 | D1056 | 41133 | 98.585 | 97.849 |
| D0167 | 34191 | 96.462 | 94.966 | D1057 | 39582 | 98.471 | 97.724 |
| D0168 | 39900 | 98.401 | 97.607 | D1058 | 33257 | 98.215 | 97.383 |
| D0169 | 36321 | 97.881 | 96.979 | D1059 | 30421 | 99.228 | 96.679 |
| D0170 | 12079 | 97.544 | 96.327 | D1060 | 68259 | 99.401 | 97.162 |
| D0171 | 37228 | 98.005 | 97.076 | D1061 | 36769 | 99.315 | 96.935 |
| D0172 | 25034 | 98.214 | 97.341 | D1062 | 39832 | 97.58 | 96.099 |
| D0173 | 35784 | 98.556 | 97.849 | D1063 | 44555 | 98.558 | 97.809 |
| D0174 | 27293 | 97.494 | 96.322 | D1064 | 53472 | 97.806 | 96.873 |
| D0175 | 37794 | 96.169 | 94.448 | D1065 | 43113 | 98.091 | 97.011 |
| D0176 | 38978 | 98.633 | 97.874 | D1066 | 42862 | 99.358 | 97.097 |
| D0177 | 40196 | 98.078 | 97.141 | D1067 | 49557 | 97.733 | 96.466 |
| D0178 | 42478 | 98.399 | 97.557 | D1068 | 37362 | 99.17 | 96.47 |
| D0179 | 47972 | 98.558 | 97.796 | D1069 | 25448 | 94.581 | 92.253 |
| D0180 | 30615 | 98.537 | 97.83 | D1070 | 39646 | 99.305 | 96.922 |
| D0181 | 33376 | 98.308 | 97.484 | D1071 | 39174 | 99.327 | 96.852 |
| D0182 | 45137 | 98.658 | 97.99 | D1072 | 45686 | 98.048 | 97.045 |
| D0183 | 26643 | 97.801 | 96.694 | D1073 | 35350 | 96.264 | 94.63 |
| D0184 | 51765 | 98.494 | 97.757 | D1074 | 41247 | 99.416 | 97.307 |
| D0185 | 45277 | 98.634 | 97.902 | D1075 | 37206 | 95.729 | 93.917 |
| D0186 | 43924 | 98.107 | 97.156 | D1076 | 48760 | 98.191 | 97.406 |
| D0187 | 40970 | 98.708 | 98.075 | D1077 | 35590 | 98.589 | 97.87 |
| D0188 | 46699 | 98.147 | 97.24 | D1078 | 40401 | 99.416 | 97.265 |
| D0189 | 45552 | 98.24 | 97.349 | D1079 | 50068 | 98.473 | 97.685 |
| D0190 | 30388 | 97.86 | 96.809 | D1080 | 42821 | 97.76 | 96.779 |
| D0191 | 27043 | 98.519 | 97.684 | D1081 | 36879 | 98.286 | 97.473 |
| D0192 | 44007 | 97.817 | 96.731 | D1082 | 43441 | 98.289 | 97.487 |
| D0193 | 40735 | 98.271 | 97.465 | D1083 | 39583 | 99.345 | 97.026 |
| D0194 | 39828 | 98.594 | 97.888 | D1084 | 40775 | 99.419 | 97.317 |
| D0195 | 31345 | 98.354 | 97.495 | D1085 | 55599 | 99.187 | 97.357 |
| D0196 | 39923 | 98.073 | 97.113 | D1086 | 36141 | 97.943 | 96.788 |
| D0197 | 29546 | 97.996 | 97.094 | D1087 | 33099 | 97.787 | 96.638 |
| D0198 | 39095 | 98.631 | 97.85 | D1088 | 37240 | 99.34 | 96.945 |
| D0199 | 39423 | 98.608 | 97.903 | D1089 | 28981 | 98.457 | 97.673 |
| D0200 | 25300 | 95.496 | 93.522 | D1090 | 46597 | 98.159 | 97.292 |
| D0201 | 36972 | 98.519 | 97.815 | D1091 | 65976 | 99.335 | 96.922 |
| D0202 | 35172 | 97.092 | 95.794 | D1092 | 55367 | 99.419 | 97.239 |
| D0203 | 43437 | 98.598 | 97.913 | D1093 | 29311 | 99.38 | 97.164 |
| D0204 | 37786 | 98.179 | 97.336 | D1094 | 37908 | 95.534 | 93.551 |
| D0205 | 45875 | 98.612 | 97.953 | D1095 | 41506 | 99.305 | 96.9 |
| D0206 | 40221 | 98.208 | 97.329 | D1096 | 38104 | 99.259 | 96.69 |
| D0207 | 36142 | 98.25 | 97.359 | D1097 | 41760 | 99.357 | 97.089 |
| D0208 | 34687 | 98.102 | 97.063 | D1098 | 38964 | 99.399 | 97.239 |
| D0209 | 54770 | 99.386 | 97.118 | D1099 | 38367 | 99.357 | 96.959 |
| D0210 | 25265 | 98.308 | 97.53 | D1100 | 37563 | 99.366 | 97.05 |
| D0211 | 39043 | 97.951 | 96.953 | D1101 | 25117 | 95.596 | 93.202 |
| D0212 | 36084 | 97.997 | 96.921 | D1102 | 45774 | 99.353 | 97.073 |
| D0213 | 34935 | 96.158 | 94.278 | D1103 | 40676 | 99.381 | 97.169 |
| D0214 | 38935 | 98.639 | 97.923 | D1104 | 42609 | 98.647 | 97.943 |
| D0215 | 41587 | 98.082 | 97.163 | D1105 | 43128 | 99.295 | 96.835 |
| D0216 | 40063 | 98.443 | 97.624 | D1106 | 33639 | 99.213 | 96.584 |
| D0217 | 43199 | 98.306 | 97.461 | D1107 | 33661 | 95.106 | 92.606 |
| D0218 | 50403 | 98.588 | 97.9 | D1108 | 39494 | 99.244 | 96.728 |
| D0219 | 49414 | 98.528 | 97.751 | D1109 | 44561 | 99.294 | 96.837 |
| D0220 | 41682 | 98.568 | 97.81 | D1110 | 37367 | 99.294 | 96.849 |
| D0221 | 43574 | 98.33 | 97.472 | D1111 | 38390 | 98.066 | 97.147 |
| D0222 | 43870 | 97.625 | 96.47 | D1112 | 42701 | 99.326 | 96.956 |
| D0223 | 40723 | 98.199 | 97.298 | D1113 | 23510 | 97.459 | 96.088 |
| D0224 | 48266 | 98.151 | 97.248 | D1114 | 40728 | 99.341 | 97.045 |
| D0225 | 40337 | 94.211 | 91.858 | D1115 | 34688 | 99.178 | 96.482 |
| D0226 | 13552 | 97.159 | 95.914 | D1116 | 41054 | 99.385 | 97.028 |
| D0227 | 36728 | 98.589 | 97.81 | D1117 | 63687 | 98.897 | 98.361 |
| D0228 | 41395 | 98.407 | 97.601 | D1118 | 61068 | 98.761 | 98.125 |
| D0229 | 41860 | 98.639 | 97.917 | D1119 | 34130 | 99.332 | 97.012 |
| D0230 | 29746 | 97.238 | 95.909 | D1120 | 36759 | 97.611 | 96.284 |
| D0231 | 45696 | 98.116 | 97.218 | D1121 | 35062 | 97.498 | 96.229 |
| D0232 | 33274 | 96.847 | 95.307 | D1122 | 45454 | 99.337 | 97.002 |
| D0233 | 49662 | 98.67 | 98.013 | D1123 | 34347 | 99.303 | 96.886 |
| D0234 | 43214 | 98.135 | 97.141 | D1124 | 66995 | 97.855 | 96.668 |
| D0235 | 44589 | 98.455 | 97.738 | D1125 | 41024 | 94.769 | 92.392 |
| D0236 | 41348 | 98.034 | 97.026 | D1126 | 33364 | 99.253 | 96.739 |
| D0237 | 52482 | 98.625 | 97.961 | D1127 | 37221 | 99.363 | 97.119 |
| D0238 | 39934 | 98.595 | 97.826 | D1128 | 36074 | 96.661 | 95.091 |
| D0239 | 22154 | 96.727 | 94.939 | D1129 | 36663 | 99.324 | 96.984 |
| D0240 | 31975 | 97.229 | 95.977 | D1130 | 32066 | 99.357 | 96.963 |
| D0241 | 28969 | 97.77 | 96.73 | D1131 | 50016 | 98.454 | 97.762 |
| D0242 | 39552 | 97.512 | 96.284 | D1132 | 34169 | 99.25 | 96.563 |
| D0243 | 38113 | 96.206 | 94.508 | D1133 | 27019 | 99.348 | 96.888 |
| D0244 | 47424 | 98.463 | 97.673 | D1134 | 39719 | 99.259 | 96.532 |
| D0245 | 38640 | 98.158 | 97.211 | D1135 | 44002 | 99.22 | 96.583 |
| D0246 | 42116 | 97.975 | 96.967 | D1136 | 44413 | 99.214 | 96.561 |
| D0247 | 45506 | 98.409 | 97.521 | D1137 | 43941 | 97.347 | 96.232 |
| D0248 | 44748 | 98.181 | 97.28 | D1138 | 37157 | 99.352 | 97.038 |
| D0249 | 43972 | 97.67 | 96.61 | D1139 | 41024 | 99.394 | 97.11 |
| D0250 | 39389 | 97.145 | 95.817 | D1140 | 40013 | 98.582 | 97.88 |
| D0251 | 39827 | 98.427 | 97.665 | D1141 | 38568 | 98.258 | 97.48 |
| D0252 | 39950 | 98.036 | 97.026 | D1142 | 40547 | 98.084 | 97.152 |
| D0253 | 33760 | 97.282 | 96.07 | D1143 | 37897 | 99.417 | 97.311 |
| D0254 | 42055 | 98.276 | 97.402 | D1144 | 51497 | 99.327 | 96.969 |
| D0255 | 42658 | 97.483 | 96.297 | D1145 | 38998 | 99.24 | 96.615 |
| D0256 | 45997 | 98.426 | 97.639 | D1146 | 41841 | 99.356 | 97.064 |
| D0257 | 31654 | 98.533 | 97.802 | D1147 | 39312 | 99.345 | 97.002 |
| D0258 | 43569 | 98.536 | 97.834 | D1148 | 36205 | 99.329 | 96.992 |
| D0259 | 41763 | 98.598 | 97.865 | D1149 | 34921 | 99.31 | 96.93 |
| D0260 | 34507 | 96.055 | 94.299 | D1150 | 29265 | 98.66 | 97.994 |
| D0261 | 39458 | 98.326 | 97.45 | D1151 | 37682 | 99.392 | 97.183 |
| D0262 | 42207 | 98.522 | 97.752 | D1152 | 34539 | 99.299 | 96.903 |
| D0263 | 36218 | 97.951 | 96.933 | D1153 | 38524 | 99.115 | 96.301 |
| D0264 | 36373 | 98.47 | 97.689 | D1154 | 17490 | 97.4 | 96.244 |
| D0265 | 29187 | 97.682 | 96.484 | D1155 | 35094 | 99.328 | 96.999 |
| D0266 | 43711 | 96.468 | 94.979 | D1156 | 31046 | 99.264 | 96.77 |
| D0267 | 50170 | 98.558 | 97.792 | D1157 | 33226 | 99.36 | 97.082 |
| D0268 | 37727 | 98.621 | 97.955 | D1158 | 53231 | 99.263 | 96.649 |
| D0269 | 48243 | 98.619 | 97.862 | D1159 | 27623 | 99.344 | 97.019 |
| D0270 | 45182 | 98.582 | 97.854 | D1160 | 57758 | 99.348 | 96.941 |
| D0271 | 47403 | 98.708 | 98.075 | D1161 | 33895 | 97.539 | 96.465 |
| D0272 | 51282 | 98.368 | 97.585 | D1162 | 42132 | 99.373 | 97.162 |
| D0273 | 42827 | 99.354 | 96.981 | D1163 | 24979 | 97.301 | 96.152 |
| D0274 | 34128 | 99.269 | 96.596 | D1164 | 33534 | 99.33 | 96.98 |
| D0275 | 47181 | 98.474 | 97.71 | D1165 | 38528 | 99.39 | 97.226 |
| D0276 | 45798 | 98.6 | 97.857 | D1166 | 33677 | 99.374 | 97.155 |
| D0277 | 43507 | 98.466 | 97.729 | D1167 | 31684 | 99.349 | 97.034 |
| D0278 | 41826 | 98.362 | 97.497 | D1168 | 32890 | 99.392 | 97.206 |
| D0279 | 40079 | 98.525 | 97.806 | D1169 | 61010 | 99.21 | 96.476 |
| D0280 | 40531 | 98.689 | 98.018 | D1170 | 34422 | 96.522 | 95.045 |
| D0281 | 53242 | 98.396 | 97.557 | D1171 | 35845 | 99.216 | 96.534 |
| D0282 | 34162 | 98.26 | 97.498 | D1172 | 34240 | 99.28 | 96.813 |
| D0283 | 29717 | 97.227 | 96.002 | D1173 | 34718 | 99.312 | 96.944 |
| D0284 | 44753 | 98.471 | 97.699 | D1174 | 34146 | 99.212 | 96.596 |
| D0285 | 40004 | 98.63 | 97.971 | D1175 | 35362 | 99.411 | 97.284 |
| D0286 | 38422 | 98.142 | 97.307 | D1176 | 33706 | 99.379 | 97.165 |
| D0287 | 33324 | 98.28 | 97.431 | D1177 | 32799 | 99.343 | 97.047 |
| D0288 | 52498 | 98.256 | 97.38 | D1178 | 36458 | 96.615 | 95.12 |
| D0289 | 29780 | 98 | 97.152 | D1179 | 60289 | 99.317 | 96.793 |
| D0290 | 30238 | 97.978 | 97.071 | D1180 | 17977 | 96.347 | 94.948 |
| D0291 | 59257 | 98.012 | 97.106 | D1181 | 33715 | 99.35 | 97.065 |
| D0292 | 47570 | 98.633 | 97.975 | D1182 | 66702 | 99.251 | 96.629 |
| D0293 | 31911 | 97.074 | 95.822 | D1183 | 38894 | 99.276 | 96.826 |
| D0294 | 48583 | 98.327 | 97.499 | D1184 | 33913 | 99.323 | 96.992 |
| D0295 | 24549 | 98.234 | 97.429 | D1185 | 37085 | 96.652 | 95.122 |
| D0296 | 42516 | 97.951 | 97.011 | D1186 | 39839 | 99.399 | 97.231 |
| D0297 | 41428 | 98.556 | 97.861 | D1187 | 9515 | 93.485 | 90.75 |
| D0298 | 42393 | 98.403 | 97.591 | D1188 | 34106 | 99.254 | 96.731 |
| D0299 | 34756 | 98.691 | 97.978 | D1189 | 37878 | 97.529 | 96.44 |
| D0300 | 42398 | 98.572 | 97.869 | D1190 | 43007 | 99.321 | 96.962 |
| D0301 | 40237 | 98.595 | 97.87 | D1191 | 36443 | 97.821 | 96.705 |
| D0302 | 38656 | 96.874 | 95.405 | D1192 | 53009 | 97.667 | 96.536 |
| D0303 | 33673 | 98.54 | 97.711 | D1193 | 40934 | 99.218 | 96.616 |
| D0304 | 42636 | 98.617 | 97.888 | D1194 | 34900 | 99.245 | 96.717 |
| D0305 | 35470 | 97.583 | 96.351 | D1195 | 40416 | 99.285 | 96.805 |
| D0306 | 44227 | 97.643 | 96.471 | D1196 | 41144 | 97.483 | 96.307 |
| D0307 | 32657 | 99.34 | 96.917 | D1197 | 48783 | 99.339 | 97.007 |
| D0308 | 46947 | 99.388 | 97.093 | D1198 | 48851 | 99.375 | 97.132 |
| D0309 | 47472 | 98.388 | 97.559 | D1199 | 43483 | 99.394 | 97.21 |
| D0310 | 42593 | 98.12 | 97.223 | D1200 | 37218 | 97.432 | 96.404 |
| D0311 | 41081 | 99.351 | 96.914 | D1201 | 35553 | 99.233 | 96.697 |
| D0312 | 50825 | 96.32 | 94.677 | D1202 | 34648 | 99.23 | 96.611 |
| D0313 | 53491 | 99.068 | 97.377 | D1203 | 58565 | 99.257 | 96.632 |
| D0314 | 46907 | 97.619 | 96.56 | D1204 | 40577 | 98.155 | 97.274 |
| D0315 | 43119 | 98.626 | 97.906 | D1205 | 44142 | 96.649 | 95.326 |
| D0316 | 48997 | 97.99 | 97.091 | D1206 | 32609 | 99.372 | 97.146 |
| D0317 | 39840 | 98.071 | 97.188 | D1207 | 34083 | 99.388 | 97.227 |
| D0318 | 34794 | 98.166 | 97.25 | D1208 | 18691 | 94.957 | 93.027 |
| D0319 | 56361 | 98.544 | 97.8 | D1209 | 39202 | 99.258 | 96.726 |
| D0320 | 44466 | 98.441 | 97.664 | D1210 | 37093 | 99.403 | 97.213 |
| D0321 | 39289 | 97.471 | 96.372 | D1211 | 33380 | 99.337 | 97.035 |
| D0322 | 40586 | 98.453 | 97.699 | D1212 | 46899 | 97.844 | 96.812 |
| D0323 | 51204 | 98.266 | 97.459 | D1213 | 36769 | 97.095 | 95.731 |
| D0324 | 48299 | 98.344 | 97.453 | D1214 | 31431 | 99.405 | 97.257 |
| D0325 | 50404 | 98.587 | 97.909 | D1215 | 43849 | 97.826 | 96.762 |
| D0326 | 37163 | 98.262 | 97.427 | D1216 | 41272 | 98.055 | 97.076 |
| D0327 | 41429 | 97.597 | 96.488 | D1217 | 68382 | 99.278 | 96.525 |
| D0328 | 46010 | 98.638 | 97.951 | D1218 | 40009 | 99.299 | 96.863 |
| D0329 | 41466 | 98.228 | 97.413 | D1219 | 28794 | 99.376 | 97.134 |
| D0330 | 36121 | 98.073 | 97.115 | D1220 | 30927 | 97.383 | 96.317 |
| D0331 | 41824 | 98.455 | 97.684 | D1221 | 39009 | 99.422 | 97.323 |
| D0332 | 30977 | 98.528 | 97.712 | D1222 | 35973 | 99.337 | 96.996 |
| D0333 | 38783 | 97.987 | 96.99 | D1223 | 34154 | 99.349 | 97.033 |
| D0334 | 36250 | 98.046 | 97.195 | D1224 | 29789 | 99.354 | 97.053 |
| D0335 | 46667 | 98.122 | 97.196 | D1225 | 42471 | 96.99 | 95.564 |
| D0336 | 43245 | 98.654 | 97.971 | D1226 | 32606 | 99.28 | 96.793 |
| D0337 | 38163 | 98.387 | 97.566 | D1227 | 57832 | 99.343 | 96.954 |
| D0338 | 32579 | 99.305 | 96.827 | D1228 | 80175 | 99.399 | 97.139 |
| D0339 | 43157 | 97.715 | 96.784 | D1229 | 33278 | 99.34 | 97.007 |
| D0340 | 47183 | 98.551 | 97.781 | D1230 | 33262 | 99.342 | 97.044 |
| D0341 | 34156 | 96.964 | 95.631 | D1231 | 36530 | 98.279 | 97.566 |
| D0342 | 47449 | 98.725 | 98.085 | D1232 | 28932 | 99.411 | 97.275 |
| D0343 | 35012 | 97.867 | 96.785 | D1233 | 38545 | 99.37 | 97.161 |
| D0344 | 42953 | 96.821 | 95.457 | D1234 | 30822 | 99.276 | 96.758 |
| D0345 | 42226 | 98.187 | 97.348 | D1235 | 34009 | 99.422 | 97.33 |
| D0346 | 68606 | 97.441 | 95.068 | D1236 | 39005 | 99.349 | 97.041 |
| D0347 | 43927 | 98.033 | 97.138 | D1237 | 80031 | 99.359 | 96.966 |
| D0348 | 42982 | 98.556 | 97.87 | D1238 | 31878 | 99.368 | 97.081 |
| D0349 | 32518 | 99.354 | 96.958 | D1239 | 51730 | 99.229 | 96.44 |
| D0350 | 42215 | 98.473 | 97.736 | D1240 | 42909 | 99.429 | 97.356 |
| D0351 | 50888 | 99.35 | 97.023 | D1241 | 40572 | 99.274 | 96.808 |
| D0352 | 30176 | 96.8 | 95.48 | D1242 | 33125 | 98.066 | 97.07 |
| D0353 | 36383 | 97.942 | 96.922 | D1243 | 32604 | 99.283 | 96.818 |
| D0354 | 46399 | 98.68 | 97.996 | D1244 | 30881 | 97.106 | 95.842 |
| D0355 | 43238 | 98.085 | 97.224 | D1245 | 44183 | 96.694 | 95.257 |
| D0356 | 41696 | 98.406 | 97.658 | D1246 | 15233 | 94.731 | 92.418 |
| D0357 | 38253 | 98.111 | 97.119 | D1247 | 46513 | 97.808 | 96.856 |
| D0358 | 37875 | 98.189 | 97.286 | D1248 | 40461 | 99.385 | 97.166 |
| D0359 | 29497 | 99.319 | 96.834 | D1249 | 38467 | 99.328 | 96.954 |
| D0360 | 39057 | 97.767 | 96.762 | D1250 | 37041 | 99.381 | 97.145 |
| D0361 | 39256 | 97.708 | 96.645 | D1251 | 41798 | 97.698 | 96.677 |
| D0362 | 46940 | 98.052 | 97.148 | D1252 | 16285 | 97.232 | 95.996 |
| D0363 | 43536 | 98.01 | 97.006 | D1253 | 41563 | 98.092 | 97.175 |
| D0364 | 31208 | 96.54 | 95.005 | D1254 | 36859 | 99.412 | 97.267 |
| D0365 | 39441 | 98.181 | 97.214 | D1255 | 41593 | 99.363 | 97.1 |
| D0366 | 33499 | 97.278 | 95.994 | D1256 | 39943 | 99.191 | 96.54 |
| D0367 | 43408 | 97.659 | 96.562 | D1257 | 33854 | 99.427 | 97.336 |
| D0368 | 46990 | 97.374 | 96.272 | D1258 | 87169 | 99.404 | 97.165 |
| D0369 | 35127 | 97.138 | 95.751 | D1259 | 32932 | 99.394 | 97.252 |
| D0370 | 44560 | 99.352 | 96.96 | D1260 | 41122 | 99.353 | 97.088 |
| D0371 | 44044 | 98.352 | 97.58 | D1261 | 36367 | 99.337 | 96.986 |
| D0372 | 48712 | 97.927 | 96.861 | D1262 | 33467 | 99.377 | 97.134 |
| D0373 | 36210 | 97.907 | 96.894 | D1263 | 33359 | 99.333 | 96.973 |
| D0374 | 45221 | 98.068 | 97.212 | D1264 | 31573 | 99.333 | 96.978 |
| D0375 | 47994 | 99.384 | 97.039 | D1265 | 30356 | 96.655 | 95.284 |
| D0376 | 43328 | 98.034 | 97.007 | D1266 | 32426 | 99.377 | 97.159 |
| D0377 | 53336 | 97.651 | 96.537 | D1267 | 37911 | 99.3 | 96.891 |
| D0378 | 41578 | 98.006 | 97.078 | D1268 | 37315 | 99.36 | 97.082 |
| D0379 | 37864 | 98.588 | 97.85 | D1269 | 32722 | 99.365 | 97.099 |
| D0380 | 45304 | 97.956 | 97.042 | D1270 | 39847 | 97.905 | 96.912 |
| D0381 | 42377 | 98.425 | 97.674 | D1271 | 51210 | 95.557 | 93.711 |
| D0382 | 40246 | 97.651 | 96.445 | D1272 | 28941 | 99.381 | 97.163 |
| D0383 | 35164 | 97.382 | 96.31 | D1273 | 38023 | 99.266 | 96.736 |
| D0384 | 40528 | 96.517 | 94.92 | D1274 | 32193 | 99.26 | 96.714 |
| D0385 | 38512 | 96.472 | 94.923 | D1275 | 39931 | 99.39 | 97.219 |
| D0386 | 35733 | 97.741 | 96.694 | D1276 | 30797 | 99.332 | 97.002 |
| D0387 | 43128 | 97.934 | 96.923 | D1277 | 30447 | 99.344 | 97.037 |
| D0388 | 37913 | 98.045 | 97.066 | D1278 | 32365 | 99.279 | 96.801 |
| D0389 | 35631 | 98.18 | 97.265 | D1279 | 28087 | 99.389 | 97.207 |
| D0390 | 41905 | 98.034 | 97.11 | D1280 | 39030 | 99.419 | 97.309 |
| D0391 | 33138 | 97.755 | 96.713 | D1281 | 33757 | 99.395 | 97.225 |
| D0392 | 32498 | 99.377 | 97.064 | D1282 | 28495 | 99.318 | 96.903 |
| D0393 | 59857 | 98.519 | 97.737 | D1283 | 46319 | 97.832 | 96.767 |
| D0394 | 40901 | 98.172 | 97.166 | D1284 | 32441 | 99.315 | 96.992 |
| D0395 | 32781 | 96.887 | 95.471 | D1285 | 54708 | 97.642 | 96.557 |
| D0396 | 36368 | 97.156 | 95.849 | D1286 | 57730 | 98.129 | 96.832 |
| D0397 | 38390 | 97.685 | 96.645 | D1287 | 33565 | 99.37 | 97.115 |
| D0398 | 23890 | 98.035 | 97.108 | D1288 | 29006 | 99.358 | 97.078 |
| D0399 | 34342 | 97.977 | 97.02 | D1289 | 31236 | 99.389 | 97.198 |
| D0400 | 38830 | 98.54 | 97.85 | D1290 | 31845 | 99.329 | 96.946 |
| D0401 | 32679 | 97.343 | 96.138 | D1291 | 44235 | 99.314 | 96.949 |
| D0402 | 33925 | 97.97 | 96.86 | D1292 | 46996 | 98.349 | 97.527 |
| D0403 | 37370 | 97.979 | 96.904 | D1293 | 64166 | 98.387 | 97.659 |
| D0404 | 36523 | 97.033 | 95.746 | D1294 | 30295 | 99.393 | 97.212 |
| D0405 | 46907 | 98.213 | 97.368 | D1295 | 57496 | 98.002 | 96.513 |
| D0406 | 33263 | 96.398 | 94.814 | D1296 | 34709 | 98.649 | 97.986 |
| D0407 | 40098 | 97.893 | 96.813 | D1297 | 35525 | 98.418 | 97.719 |
| D0408 | 34682 | 97.34 | 96.132 | D1298 | 32638 | 99.388 | 97.187 |
| D0409 | 36641 | 98.12 | 97.213 | D1299 | 40409 | 96.139 | 94.55 |
| D0410 | 35134 | 97.554 | 96.346 | D1300 | 51540 | 98.151 | 97.316 |
| D0411 | 43027 | 98.046 | 97.138 | D1301 | 35586 | 99.36 | 97.091 |
| D0412 | 42882 | 96.633 | 95.077 | D1302 | 37873 | 97.894 | 96.886 |
| D0413 | 52417 | 98.515 | 97.755 | D1303 | 38481 | 99.41 | 97.272 |
| D0414 | 36751 | 99.393 | 97.2 | D1304 | 39218 | 99.367 | 97.089 |
| D0415 | 42282 | 98.136 | 97.176 | D1305 | 31038 | 99.379 | 97.16 |
| D0416 | 43325 | 97.846 | 96.765 | D1306 | 32424 | 99.394 | 97.239 |
| D0417 | 29552 | 96.716 | 95.125 | D1307 | 39998 | 97.424 | 96.348 |
| D0418 | 45440 | 98.362 | 97.569 | D1308 | 27475 | 99.396 | 97.21 |
| D0419 | 48023 | 97.642 | 96.592 | D1309 | 34564 | 99.24 | 96.673 |
| D0420 | 34389 | 97.9 | 96.858 | D1310 | 29698 | 99.372 | 97.132 |
| D0421 | 41328 | 98.277 | 97.341 | D1311 | 38664 | 98.257 | 97.447 |
| D0422 | 44369 | 98.081 | 97.024 | D1312 | 35902 | 99.398 | 97.192 |
| D0423 | 42721 | 97.826 | 96.736 | D1313 | 73965 | 98.381 | 97.71 |
| D0424 | 39527 | 97.971 | 97.034 | D1314 | 36783 | 99.373 | 97.148 |
| D0425 | 47797 | 98.051 | 97.17 | D1315 | 40379 | 98.711 | 98.113 |
| D0426 | 34475 | 97.318 | 95.983 | D1316 | 32848 | 99.394 | 97.24 |
| D0427 | 39943 | 98.294 | 97.554 | D1317 | 34436 | 99.324 | 96.947 |
| D0428 | 31663 | 97.257 | 95.933 | D1318 | 25217 | 97.087 | 95.81 |
| D0429 | 38401 | 96.579 | 94.989 | D1319 | 29068 | 99.257 | 96.708 |
| D0430 | 31883 | 96.586 | 95.094 | D1320 | 35085 | 99.352 | 97.078 |
| D0431 | 46128 | 98.695 | 97.958 | D1321 | 24224 | 93.738 | 91.282 |
| D0432 | 41246 | 97.465 | 96.167 | D1322 | 33459 | 99.345 | 97.033 |
| D0433 | 46118 | 98.183 | 97.279 | D1323 | 31623 | 99.384 | 97.177 |
| D0434 | 38977 | 99.352 | 96.902 | D1324 | 39469 | 99.329 | 96.976 |
| D0435 | 45926 | 99.393 | 97.124 | D1325 | 30146 | 99.37 | 97.143 |
| D0436 | 32323 | 98.328 | 97.496 | D1326 | 31474 | 99.37 | 97.135 |
| D0437 | 28600 | 97.82 | 96.698 | D1327 | 41808 | 97.782 | 96.746 |
| D0438 | 39669 | 97.757 | 96.632 | D1328 | 27962 | 99.425 | 97.351 |
| D0439 | 31359 | 97.878 | 96.943 | D1329 | 34036 | 97.452 | 96.395 |
| D0440 | 39014 | 98.081 | 97.08 | D1330 | 25244 | 99.42 | 97.295 |
| D0441 | 31204 | 99.309 | 96.798 | D1331 | 31972 | 98.768 | 98.156 |
| D0442 | 32782 | 97.957 | 96.943 | D1332 | 53080 | 98.705 | 98.086 |
| D0443 | 39933 | 98.57 | 97.86 | D1333 | 40740 | 99.363 | 97.101 |
| D0444 | 44063 | 97.997 | 96.954 | D1334 | 52730 | 98.506 | 96.362 |
| D0445 | 37260 | 97.263 | 96.058 | D1335 | 41692 | 97.364 | 96.139 |
| D0446 | 38807 | 97.975 | 96.863 | D1336 | 43305 | 97.867 | 96.759 |
| D0447 | 34312 | 97.974 | 97.044 | D1337 | 26879 | 98.278 | 97.541 |
| D0448 | 39024 | 97.98 | 97.054 | D1338 | 49960 | 98.03 | 97.152 |
| D0449 | 39103 | 98.121 | 97.233 | D1339 | 52092 | 98.139 | 97.266 |
| D0450 | 41688 | 95.841 | 93.943 | D1340 | 30800 | 96.449 | 95.002 |
| D0451 | 45641 | 97.821 | 96.75 | D1341 | 15955 | 95.248 | 93.252 |
| D0452 | 40986 | 97.258 | 95.853 | D1342 | 29665 | 99.356 | 97.088 |
| D0453 | 46169 | 98.139 | 97.154 | D1343 | 40878 | 99.326 | 96.992 |
| D0454 | 38687 | 98.144 | 97.265 | D1344 | 54256 | 97.748 | 96.607 |
| D0455 | 39688 | 97.531 | 96.34 | D1345 | 43739 | 96.079 | 94.385 |
| D0456 | 29134 | 97.361 | 96.138 | D1346 | 44062 | 98.095 | 97.124 |
| D0457 | 44231 | 97.914 | 96.855 | D1347 | 56378 | 98.896 | 97.218 |
| D0458 | 32219 | 96.955 | 95.595 | D1348 | 34598 | 99.328 | 96.953 |
| D0459 | 38903 | 99.371 | 97.137 | D1349 | 38346 | 96.639 | 95.155 |
| D0460 | 65313 | 99.372 | 97.025 | D1350 | 33985 | 98.76 | 98.083 |
| D0461 | 45904 | 97.71 | 96.418 | D1351 | 37359 | 99.33 | 96.961 |
| D0462 | 59475 | 97.824 | 96.825 | D1352 | 39360 | 99.419 | 97.325 |
| D0463 | 49884 | 97.945 | 96.977 | D1353 | 34964 | 98.106 | 97.254 |
| D0464 | 78922 | 99.392 | 97.077 | D1354 | 37229 | 96.425 | 94.959 |
| D0465 | 33118 | 97.556 | 96.441 | D1355 | 30284 | 99.414 | 97.304 |
| D0466 | 28158 | 96.805 | 95.223 | D1356 | 50703 | 98.068 | 97.222 |
| D0467 | 33273 | 97.574 | 96.432 | D1357 | 29561 | 98.25 | 97.382 |
| D0468 | 27089 | 96.842 | 95.419 | D1358 | 33404 | 99.357 | 97.127 |
| D0469 | 39489 | 98.014 | 96.934 | D1359 | 59499 | 97.447 | 96.4 |
| D0470 | 43450 | 97.32 | 96.017 | D1360 | 43141 | 97.93 | 97.017 |
| D0471 | 35759 | 97.815 | 96.767 | D1361 | 33527 | 97.361 | 96.219 |
| D0472 | 30254 | 98.103 | 97.111 | D1362 | 40448 | 98.123 | 97.256 |
| D0473 | 28751 | 99.37 | 97.046 | D1363 | 50784 | 98.018 | 97.141 |
| D0474 | 41771 | 99.342 | 96.871 | D1364 | 42999 | 97.561 | 96.477 |
| D0475 | 55654 | 98.532 | 97.829 | D1365 | 41043 | 95.945 | 94.208 |
| D0476 | 41915 | 99.356 | 96.898 | D1366 | 50852 | 95.534 | 93.821 |
| D0477 | 38523 | 97.299 | 96.052 | D1367 | 42872 | 97.94 | 96.901 |
| D0478 | 32003 | 98.128 | 97.157 | D1368 | 35591 | 98.036 | 97.08 |
| D0479 | 37140 | 98.167 | 97.282 | D1369 | 47001 | 97.755 | 96.731 |
| D0480 | 48271 | 99.406 | 97.252 | D1370 | 46509 | 98.187 | 97.284 |
| D0481 | 34911 | 98.09 | 97.09 | D1371 | 35791 | 97.669 | 96.589 |
| D0482 | 47529 | 98.076 | 97.124 | D1372 | 41137 | 97.421 | 96.33 |
| D0483 | 39941 | 98.198 | 97.15 | D1373 | 54236 | 98.763 | 98.113 |
| D0484 | 41722 | 98.018 | 97.093 | D1374 | 54763 | 97.991 | 97.063 |
| D0485 | 39927 | 97.179 | 95.923 | D1375 | 40069 | 99.318 | 96.927 |
| D0486 | 45687 | 98.661 | 97.965 | D1376 | 48104 | 97.002 | 95.704 |
| D0487 | 24696 | 99.393 | 97.128 | D1377 | 28294 | 98.199 | 97.32 |
| D0488 | 43085 | 98.214 | 97.298 | D1378 | 45809 | 98.228 | 97.323 |
| D0489 | 35626 | 99.365 | 97.091 | D1379 | 45803 | 98.139 | 97.244 |
| D0490 | 37530 | 98.01 | 97.008 | D1380 | 46253 | 96.753 | 95.413 |
| D0491 | 45444 | 98.039 | 97.051 | D1381 | 54232 | 97.914 | 97.003 |
| D0492 | 28301 | 97.61 | 96.492 | D1382 | 31047 | 97.788 | 96.723 |
| D0493 | 33565 | 97.044 | 95.74 | D1383 | 34850 | 98.26 | 97.412 |
| D0494 | 43173 | 98.597 | 97.841 | D1384 | 42574 | 95.436 | 93.549 |
| D0495 | 52682 | 99.36 | 97.06 | D1385 | 48550 | 97.338 | 96.131 |
| D0496 | 41742 | 98.19 | 97.247 | D1386 | 33117 | 96.684 | 95.275 |
| D0497 | 53936 | 97.981 | 97.052 | D1387 | 32499 | 96.485 | 94.961 |
| D0498 | 34895 | 98.191 | 97.251 | D1388 | 35230 | 99.35 | 97.055 |
| D0499 | 45663 | 98.427 | 97.68 | D1389 | 37301 | 98.155 | 97.237 |
| D0500 | 33048 | 99.331 | 96.88 | D1390 | 50302 | 97.444 | 96.287 |
| D0501 | 35960 | 95.683 | 93.687 | D1391 | 39232 | 99.332 | 96.952 |
| D0502 | 37569 | 98.104 | 97.133 | D1392 | 34096 | 99.385 | 97.188 |
| D0503 | 36368 | 97.785 | 96.745 | D1393 | 46291 | 97.363 | 96.27 |
| D0504 | 49095 | 97.901 | 96.781 | D1394 | 40385 | 97.881 | 96.935 |
| D0505 | 37530 | 98.163 | 97.194 | D1395 | 46519 | 96.538 | 95.071 |
| D0506 | 29547 | 97.426 | 96.233 | D1396 | 49336 | 97.74 | 96.767 |
| D0507 | 43212 | 97.449 | 96.321 | D1397 | 44090 | 97.788 | 96.776 |
| D0508 | 33319 | 97.539 | 96.406 | D1398 | 46595 | 97.866 | 96.831 |
| D0509 | 32222 | 97.561 | 96.323 | D1399 | 38996 | 97.886 | 96.928 |
| D0510 | 39375 | 97.66 | 96.506 | D1400 | 32194 | 98.563 | 97.819 |
| D0511 | 34456 | 97.444 | 96.13 | D1401 | 51389 | 99.199 | 97.397 |
| D0512 | 37455 | 98.19 | 97.208 | D1402 | 31774 | 96.935 | 95.646 |
| D0513 | 36907 | 99.368 | 97.02 | D1403 | 47874 | 97.388 | 96.235 |
| D0514 | 40080 | 96 | 94.182 | D1404 | 44478 | 98.19 | 97.261 |
| D0515 | 37797 | 97.532 | 96.339 | D1405 | 45905 | 98.089 | 97.153 |
| D0516 | 47443 | 98.429 | 97.592 | D1406 | 30722 | 97.388 | 96.271 |
| D0517 | 50980 | 98.094 | 97.174 | D1407 | 47827 | 97.955 | 97.019 |
| D0518 | 40150 | 99.33 | 96.94 | D1408 | 45316 | 97.156 | 95.909 |
| D0519 | 48228 | 99.413 | 97.253 | D1409 | 33448 | 95.802 | 94.157 |
| D0520 | 32593 | 99.383 | 97.185 | D1410 | 46829 | 97.965 | 97.027 |
| D0521 | 30528 | 97.442 | 96.157 | D1411 | 48470 | 97.401 | 96.258 |
| D0522 | 43836 | 98.124 | 97.203 | D1412 | 35760 | 97.716 | 96.55 |
| D0523 | 35947 | 97.728 | 96.64 | D1413 | 48685 | 98.082 | 97.172 |
| D0524 | 45551 | 98.057 | 97.051 | D1414 | 37330 | 98.587 | 97.918 |
| D0525 | 38491 | 97.575 | 96.469 | D1415 | 31189 | 98.755 | 98.074 |
| D0526 | 42828 | 97.935 | 96.817 | D1416 | 33532 | 99.374 | 97.16 |
| D0527 | 45719 | 98.135 | 97.16 | D1417 | 49397 | 97.741 | 96.756 |
| D0528 | 32428 | 96.984 | 95.502 | D1418 | 42373 | 95.446 | 93.425 |
| D0529 | 34534 | 97.446 | 96.142 | D1419 | 21074 | 94.598 | 92.332 |
| D0530 | 41813 | 99.31 | 96.884 | D1420 | 30568 | 98.124 | 97.227 |
| D0531 | 48883 | 98.152 | 97.182 | D1421 | 37426 | 97.973 | 97.01 |
| D0532 | 45718 | 97.876 | 96.829 | D1422 | 39728 | 99.351 | 97.082 |
| D0533 | 38489 | 97.487 | 96.332 | D1423 | 49539 | 97.254 | 96.115 |
| D0534 | 42990 | 98.026 | 97.019 | D1424 | 40072 | 97.161 | 95.978 |
| D0535 | 60249 | 99.372 | 97.021 | D1425 | 33293 | 99.367 | 97.123 |
| D0536 | 47491 | 99.243 | 96.587 | D1426 | 48677 | 97.865 | 96.916 |
| D0537 | 37261 | 96.773 | 95.353 | D1427 | 29635 | 96.812 | 95.349 |
| D0538 | 38816 | 94.65 | 92.236 | D1428 | 46956 | 97.232 | 95.926 |
| D0539 | 38329 | 98.041 | 96.983 | D1429 | 54333 | 97.993 | 97.022 |
| D0540 | 41353 | 98.049 | 97.121 | D1430 | 48872 | 98.276 | 97.445 |
| D0541 | 43805 | 98.218 | 97.357 | D1431 | 44413 | 98.219 | 97.328 |
| D0542 | 42801 | 99.303 | 96.735 | D1432 | 43982 | 98.152 | 97.198 |
| D0543 | 32942 | 98.227 | 97.406 | D1433 | 43940 | 96.046 | 94.348 |
| D0544 | 45509 | 98.055 | 97.075 | D1434 | 49457 | 97.584 | 96.554 |
| D0545 | 64368 | 99.261 | 96.648 | D1435 | 39961 | 97.967 | 97.061 |
| D0546 | 66899 | 99.405 | 97.178 | D1436 | 43503 | 97.143 | 95.945 |
| D0547 | 59128 | 96.737 | 95.352 | D1437 | 45474 | 99.365 | 97.134 |
| D0548 | 37351 | 99.365 | 97.008 | D1438 | 49684 | 97.392 | 96.186 |
| D0549 | 40496 | 99.387 | 97.084 | D1439 | 64441 | 97.964 | 96.93 |
| D0550 | 34708 | 97.288 | 96.014 | D1440 | 16690 | 97.349 | 95.964 |
| D0551 | 46069 | 97.923 | 96.992 | D1441 | 37293 | 95.085 | 93.101 |
| D0552 | 41376 | 97.75 | 96.641 | D1442 | 40132 | 98.211 | 97.308 |
| D0553 | 41126 | 96.887 | 95.183 | D1443 | 17483 | 97.653 | 96.519 |
| D0554 | 35920 | 99.356 | 97.003 | D1444 | 39585 | 97.878 | 96.907 |
| D0555 | 39962 | 98.056 | 97.127 | D1445 | 33857 | 96.307 | 94.798 |
| D0556 | 42386 | 97.737 | 96.712 | D1446 | 41380 | 97.979 | 97.03 |
| D0557 | 44700 | 97.4 | 96.157 | D1447 | 28687 | 99.262 | 96.678 |
| D0558 | 13328 | 97.495 | 96.278 | D1448 | 48924 | 98.706 | 98.057 |
| D0559 | 42505 | 97.93 | 96.717 | D1449 | 44412 | 97.161 | 95.909 |
| D0560 | 35327 | 99.39 | 97.093 | D1450 | 39405 | 97.071 | 95.688 |
| D0561 | 47962 | 98.24 | 97.31 | D1451 | 14233 | 97.427 | 96.249 |
| D0562 | 27936 | 97.672 | 96.58 | D1452 | 34255 | 97.886 | 96.863 |
| D0563 | 34780 | 99.356 | 97.097 | D1453 | 56380 | 98.219 | 97.385 |
| D0564 | 48657 | 99.35 | 97.057 | D1454 | 52970 | 98.233 | 97.404 |
| D0565 | 38856 | 97.183 | 95.956 | D1455 | 34656 | 98.037 | 97.144 |
| D0566 | 43569 | 98.016 | 97.065 | D1456 | 52491 | 98.148 | 97.282 |
| D0567 | 39119 | 97.875 | 96.806 | D1457 | 38287 | 98.084 | 97.007 |
| D0568 | 34224 | 97.604 | 96.501 | D1458 | 35438 | 96.908 | 95.506 |
| D0569 | 45118 | 97.81 | 96.756 | D1459 | 64641 | 98.016 | 97.159 |
| D0570 | 43206 | 97.288 | 95.987 | D1460 | 50601 | 97.225 | 96.047 |
| D0571 | 42700 | 98.654 | 97.961 | D1461 | 34992 | 96.336 | 94.851 |
| D0572 | 40726 | 96.547 | 95.115 | D1462 | 46255 | 99.084 | 97.195 |
| D0573 | 48872 | 97.926 | 96.774 | D1463 | 42873 | 97.177 | 95.856 |
| D0574 | 33909 | 98.129 | 97.143 | D1464 | 54286 | 97.776 | 96.691 |
| D0575 | 44447 | 98.527 | 97.565 | D1465 | 34446 | 98.108 | 97.271 |
| D0576 | 33992 | 97.703 | 96.539 | D1466 | 38969 | 98.005 | 96.997 |
| D0577 | 42100 | 98.119 | 97.175 | D1467 | 48528 | 97.305 | 96.083 |
| D0578 | 50414 | 98.146 | 97.189 | D1468 | 50436 | 97.874 | 96.869 |
| D0579 | 48345 | 99.387 | 97.13 | D1469 | 43553 | 97.518 | 96.471 |
| D0580 | 32429 | 99.349 | 97.057 | D1470 | 28024 | 99.369 | 97.138 |
| D0581 | 55359 | 97.302 | 96.041 | D1471 | 37302 | 97.853 | 96.771 |
| D0582 | 27057 | 99.321 | 96.848 | D1472 | 45393 | 97.814 | 96.851 |
| D0583 | 42634 | 98.109 | 97.144 | D1473 | 66867 | 97.775 | 96.739 |
| D0584 | 35895 | 99.279 | 96.654 | D1474 | 12347 | 97.532 | 96.287 |
| D0585 | 26899 | 99.434 | 97.271 | D1475 | 28246 | 99.349 | 97.024 |
| D0586 | 44994 | 98.092 | 97.145 | D1476 | 42077 | 99.167 | 97.367 |
| D0587 | 44068 | 98.169 | 97.156 | D1477 | 17583 | 97.687 | 96.547 |
| D0588 | 131 | 98.389 | 97.596 | D1478 | 51043 | 98.293 | 97.526 |
| D0589 | 30322 | 99.335 | 96.904 | D1479 | 46874 | 98.598 | 97.9 |
| D0590 | 27697 | 99.378 | 97.049 | D1480 | 39098 | 97.889 | 96.958 |
| D0591 | 44394 | 99.361 | 97.016 | D1481 | 57481 | 97.831 | 96.808 |
| D0592 | 26088 | 99.359 | 96.998 | D1482 | 12076 | 97.497 | 96.117 |
| D0593 | 39983 | 98.036 | 97.087 | D1483 | 55126 | 98.03 | 97.115 |
| D0594 | 41457 | 97.545 | 96.402 | D1484 | 60597 | 98.224 | 97.371 |
| D0595 | 40445 | 99.381 | 97.059 | D1485 | 64628 | 97.774 | 96.73 |
| D0596 | 43152 | 99.305 | 96.875 | D1486 | 40128 | 98.121 | 97.277 |
| D0597 | 31725 | 99.398 | 97.124 | D1487 | 37352 | 95.332 | 93.465 |
| D0598 | 38403 | 97.461 | 96.132 | D1488 | 70073 | 98.259 | 97.521 |
| D0599 | 43892 | 99.382 | 97.177 | D1489 | 36532 | 98.437 | 97.733 |
| D0600 | 32438 | 99.4 | 97.143 | D1490 | 49978 | 97.845 | 96.749 |
| D0601 | 44009 | 99.39 | 97.098 | D1491 | 39109 | 98.783 | 98.188 |
| D0602 | 37884 | 99.328 | 96.881 | D1492 | 49057 | 98 | 97.099 |
| D0603 | 42718 | 98.209 | 97.334 | D1493 | 48346 | 98.214 | 97.387 |
| D0604 | 29522 | 99.38 | 97.048 | D1494 | 38694 | 98.209 | 97.331 |
| D0605 | 49633 | 99.35 | 97.05 | D1495 | 40898 | 97.39 | 96.309 |
| D0606 | 38118 | 98.352 | 97.457 | D1496 | 41298 | 97.283 | 96.142 |
| D0607 | 44010 | 99.376 | 97.036 | D1497 | 48487 | 98.247 | 97.406 |
| D0608 | 44529 | 97.581 | 96.43 | D1498 | 37862 | 98.621 | 97.968 |
| D0609 | 66286 | 97.844 | 96.222 | D1499 | 56324 | 97.627 | 96.579 |
| D0610 | 43191 | 99.319 | 96.843 | D1500 | 34398 | 96.712 | 95.325 |
| D0611 | 37755 | 99.374 | 97.004 | D1501 | 30064 | 98.207 | 97.422 |
| D0612 | 39798 | 98.022 | 97.069 | D1502 | 53079 | 98.135 | 97.245 |
| D0613 | 46471 | 98.312 | 97.489 | D1503 | 39790 | 97.985 | 96.982 |
| D0614 | 40148 | 98.086 | 97.036 | D1504 | 52667 | 98.081 | 97.216 |
| D0615 | 37268 | 97.418 | 96.22 | D1505 | 51922 | 96.422 | 94.828 |
| D0616 | 49342 | 99.326 | 96.842 | D1506 | 47211 | 98.039 | 97.116 |
| D0617 | 37082 | 96.96 | 95.563 | D1507 | 49233 | 96.982 | 95.609 |
| D0618 | 26497 | 99.322 | 96.863 | D1508 | 24146 | 97.868 | 96.924 |
| D0619 | 44904 | 98.073 | 97.128 | D1509 | 42768 | 96.58 | 95.05 |
| D0620 | 48933 | 98.014 | 96.97 | D1510 | 16539 | 97.398 | 96.058 |
| D0621 | 48172 | 99.393 | 97.114 | D1511 | 51343 | 98.244 | 97.384 |
| D0622 | 31220 | 99.303 | 96.819 | D1512 | 31091 | 99.436 | 97.387 |
| D0623 | 37668 | 97.75 | 96.663 | D1513 | 41957 | 97.013 | 95.807 |
| D0624 | 40671 | 99.395 | 97.094 | D1514 | 41964 | 98.019 | 97.034 |
| D0625 | 25916 | 96.294 | 94.865 | D1515 | 36357 | 98.71 | 98.075 |
| D0626 | 29438 | 99.366 | 97.008 | D1516 | 26063 | 98.854 | 98.314 |
| D0627 | 38738 | 97.665 | 96.665 | D1517 | 22483 | 97.493 | 96.238 |
| D0628 | 42476 | 99.376 | 97.049 | D1518 | 25528 | 98.629 | 98.025 |
| D0629 | 40653 | 99.401 | 97.138 | D1519 | 42412 | 98.012 | 97.109 |
| D0630 | 31466 | 97.951 | 96.978 | D1520 | 46527 | 98.237 | 97.476 |
| D0631 | 43617 | 99.356 | 96.955 | D1521 | 54309 | 98.583 | 97.852 |
| D0632 | 48022 | 99.387 | 97.1 | D1522 | 57487 | 98.861 | 97.046 |
| D0633 | 47480 | 99.394 | 97.119 | D1523 | 47942 | 98.154 | 97.16 |
| D0634 | 26489 | 99.386 | 97.06 | D1524 | 29325 | 98.85 | 98.237 |
| D0635 | 66695 | 99.411 | 97.2 | D1525 | 32944 | 98.568 | 97.918 |
| D0636 | 45575 | 99.31 | 96.816 | D1526 | 63117 | 98.101 | 97.159 |
| D0637 | 35527 | 99.395 | 97.115 | D1527 | 37004 | 95.588 | 93.704 |
| D0638 | 39504 | 99.378 | 97.082 | D1528 | 54414 | 97.751 | 96.661 |
| D0639 | 35554 | 99.098 | 96.122 | D1529 | 40952 | 97.502 | 96.252 |
| D0640 | 38751 | 97.964 | 96.972 | D1530 | 53786 | 97.603 | 96.43 |
| D0641 | 32305 | 99.231 | 96.554 | D1531 | 79408 | 98.685 | 98.029 |
| D0642 | 50989 | 99.376 | 97.015 | D1532 | 76682 | 98.693 | 98.054 |
| D0643 | 58828 | 99.382 | 97.143 | D1533 | 41767 | 96.339 | 94.88 |
| D0644 | 30646 | 99.331 | 96.883 | D1534 | 32002 | 98.824 | 98.22 |
| D0645 | 36951 | 99.384 | 97.101 | D1535 | 59264 | 97.844 | 96.855 |
| D0646 | 34066 | 99.326 | 96.785 | D1536 | 53308 | 97.742 | 96.717 |
| D0647 | 48833 | 99.283 | 96.715 | D1537 | 49207 | 97.01 | 95.653 |
| D0648 | 43500 | 99.307 | 96.819 | D1538 | 51971 | 98.016 | 97.169 |
| D0649 | 41749 | 99.351 | 96.921 | D1539 | 32231 | 97.903 | 96.963 |
| D0650 | 31787 | 99.381 | 97.058 | D1540 | 46153 | 98.097 | 97.247 |
| D0651 | 53706 | 99.343 | 96.913 | D1541 | 44360 | 95.673 | 93.934 |
| D0652 | 45593 | 99.378 | 97.07 | D1542 | 48530 | 98.649 | 98.008 |
| D0653 | 33530 | 99.36 | 96.994 | D1543 | 57318 | 97.548 | 95.53 |
| D0654 | 46277 | 99.31 | 96.916 | D1544 | 36825 | 98.367 | 97.592 |
| D0655 | 45765 | 98.61 | 97.928 | D1545 | 24140 | 98.242 | 97.506 |
| D0656 | 31484 | 99.361 | 96.974 | D1546 | 32763 | 97.747 | 96.719 |
| D0657 | 32189 | 99.342 | 96.898 | D1547 | 27376 | 98.289 | 97.539 |
| D0658 | 31190 | 99.38 | 97.047 | D1548 | 37530 | 98.463 | 97.607 |
| D0659 | 53699 | 99.407 | 97.172 | D1549 | 56526 | 99.055 | 97.322 |
| D0660 | 41428 | 99.29 | 96.77 | D1550 | 39342 | 97.367 | 96.24 |
| D0661 | 34844 | 97.428 | 96.397 | D1551 | 38736 | 98.213 | 97.206 |
| D0662 | 44121 | 99.347 | 96.974 | D1552 | 77338 | 98.34 | 97.521 |
| D0663 | 70920 | 99.373 | 97.025 | D1553 | 60615 | 97.445 | 95.276 |
| D0664 | 50750 | 99.096 | 96.151 | D1554 | 30622 | 98.479 | 97.754 |
| D0665 | 52147 | 98.52 | 97.746 | D1555 | 41918 | 98.005 | 97.111 |
| D0666 | 60083 | 99.372 | 97.126 | D1556 | 50867 | 99.255 | 97.18 |
| D0667 | 40575 | 99.407 | 97.156 | D1557 | 39517 | 98.403 | 97.637 |
| D0668 | 26868 | 99.394 | 97.082 | D1558 | 59999 | 98.467 | 97.798 |
| D0669 | 61861 | 99.291 | 96.73 | D1559 | 49673 | 96.652 | 94.239 |
| D0670 | 44825 | 98.238 | 97.391 | D1560 | 49989 | 98.249 | 97.402 |
| D0671 | 37920 | 98.359 | 97.556 | D1561 | 53438 | 98.822 | 98.205 |
| D0672 | 33014 | 99.308 | 96.803 | D1562 | 33166 | 97.969 | 97.059 |
| D0673 | 32210 | 99.296 | 96.741 | D1563 | 39630 | 96.793 | 95.043 |
| D0674 | 44034 | 99.378 | 97.046 | D1564 | 30955 | 95.32 | 93.534 |
| D0675 | 48498 | 99.28 | 96.694 | D1565 | 39062 | 98.693 | 98.047 |
| D0676 | 44199 | 99.358 | 96.973 | D1566 | 24404 | 99.37 | 97.108 |
| D0677 | 34976 | 97.025 | 95.604 | D1567 | 38191 | 98.446 | 97.757 |
| D0678 | 54450 | 98.075 | 97.097 | D1568 | 54171 | 99.092 | 97.49 |
| D0679 | 38792 | 99.386 | 97.1 | D1569 | 41220 | 98.377 | 97.61 |
| D0680 | 80343 | 99.362 | 97.007 | D1570 | 45936 | 98.175 | 97.335 |
| D0681 | 63626 | 99.33 | 96.822 | D1571 | 38788 | 97.976 | 96.961 |
| D0682 | 43820 | 98.178 | 97.278 | D1572 | 54226 | 97.521 | 96.44 |
| D0683 | 32919 | 97.312 | 96.081 | D1573 | 44956 | 98.197 | 97.353 |
| D0684 | 43637 | 97.143 | 95.795 | D1574 | 50274 | 98.334 | 97.542 |
| D0685 | 35209 | 99.4 | 97.135 | D1575 | 64039 | 98.381 | 97.632 |
| D0686 | 65266 | 99.366 | 96.99 | D1576 | 23738 | 97.167 | 95.692 |
| D0687 | 33306 | 99.29 | 96.791 | D1577 | 49537 | 95.361 | 93.586 |
| D0688 | 32871 | 99.333 | 97.004 | D1578 | 29582 | 95.112 | 93.11 |
| D0689 | 60951 | 99.321 | 96.836 | D1579 | 34056 | 97.389 | 95.973 |
| D0690 | 67569 | 99.402 | 97.153 | D1580 | 47522 | 97.965 | 97.082 |
| D0691 | 26230 | 99.138 | 96.268 | D1581 | 45292 | 97.477 | 96.411 |
| D0692 | 69898 | 99.404 | 97.15 | D1582 | 86721 | 97.663 | 96.361 |
| D0693 | 49646 | 99.341 | 96.947 | D1583 | 28642 | 98.179 | 97.393 |
| D0694 | 25957 | 99.411 | 97.175 | D1584 | 58640 | 98.259 | 97.483 |
| D0695 | 32242 | 99.31 | 96.895 | D1585 | 48852 | 98.17 | 97.244 |
| D0696 | 40147 | 97.608 | 96.487 | D1586 | 34260 | 97.858 | 96.86 |
| D0697 | 40214 | 97.908 | 96.903 | D1587 | 42852 | 98.863 | 98.266 |
| D0698 | 39966 | 99.332 | 96.832 | D1588 | 43018 | 98.762 | 98.119 |
| D0699 | 67616 | 99.306 | 96.781 | D1589 | 38578 | 99.073 | 97.032 |
| D0700 | 44923 | 97.549 | 96.49 | D1590 | 61173 | 98.83 | 98.169 |
| D0701 | 62626 | 99.359 | 96.96 | D1591 | 54749 | 97.803 | 96.852 |
| D0702 | 62929 | 99.363 | 97.007 | D1592 | 25338 | 97.461 | 96.294 |
| D0703 | 27298 | 99.29 | 96.717 | D1593 | 58519 | 98.535 | 97.816 |
| D0704 | 36772 | 98.313 | 97.437 | D1594 | 52931 | 96.362 | 94.915 |
| D0705 | 71708 | 99.361 | 97.011 | D1595 | 20627 | 99.27 | 96.852 |
| D0706 | 56068 | 99.361 | 96.954 | D1596 | 72104 | 98.257 | 96.861 |
| D0707 | 58179 | 99.176 | 96.384 | D1597 | 31913 | 99.234 | 96.639 |
| D0708 | 61739 | 99.291 | 96.83 | D1598 | 48629 | 98.587 | 97.881 |
| D0709 | 43661 | 99.313 | 96.857 | D1599 | 35291 | 98.649 | 98.038 |
| D0710 | 64200 | 99.255 | 96.653 | D1600 | 19335 | 97.645 | 96.512 |
| D0711 | 31872 | 99.264 | 96.765 | D1601 | 65207 | 95.562 | 93.625 |
| D0712 | 37727 | 98.292 | 97.412 | D1602 | 25220 | 99.23 | 96.608 |
| D0713 | 37759 | 99.304 | 96.795 | D1603 | 43459 | 97.515 | 96.448 |
| D0714 | 28560 | 96.791 | 95.259 | D1604 | 46357 | 97.898 | 96.838 |
| D0715 | 56463 | 99.144 | 96.244 | D1605 | 27500 | 99.367 | 97.153 |
| D0716 | 61695 | 99.384 | 97.067 | D1606 | 37390 | 98.79 | 98.167 |
| D0717 | 40854 | 99.354 | 97.086 | D1607 | 30248 | 97.955 | 96.988 |
| D0718 | 33956 | 99.328 | 96.959 | D1608 | 51474 | 98.767 | 97.034 |
| D0719 | 34045 | 99.302 | 96.788 | D1609 | 34528 | 98.099 | 97.11 |
| D0720 | 36303 | 99.364 | 96.972 | D1610 | 41627 | 98.901 | 98.306 |
| D0721 | 30001 | 99.393 | 97.101 | D1611 | 27178 | 98.682 | 98.085 |
| D0722 | 68068 | 99.385 | 97.076 | D1612 | 33360 | 97.604 | 96.566 |
| D0723 | 55453 | 99.372 | 97.038 | D1613 | 28967 | 99.419 | 97.307 |
| D0724 | 40655 | 99.195 | 96.544 | D1614 | 43927 | 97.806 | 96.63 |
| D0725 | 73576 | 99.376 | 97.07 | D1615 | 47664 | 98.188 | 97.348 |
| D0726 | 41934 | 99.411 | 97.216 | D1616 | 23832 | 99.378 | 97.194 |
| D0727 | 52724 | 99.358 | 96.961 | D1617 | 52106 | 99.055 | 97.004 |
| D0728 | 73052 | 99.359 | 96.942 | D1618 | 59412 | 97.777 | 96.768 |
| D0729 | 34276 | 99.373 | 97.039 | D1619 | 53774 | 98.541 | 96.701 |
| D0730 | 58666 | 99.351 | 96.963 | D1620 | 30058 | 98.753 | 98.154 |
| D0731 | 45051 | 99.368 | 97.01 | D1621 | 52778 | 97.485 | 96.388 |
| D0732 | 86000 | 99.331 | 96.816 | D1622 | 39781 | 98.618 | 97.986 |
| D0733 | 65845 | 99.134 | 96.221 | D1623 | 24004 | 98.475 | 97.748 |
| D0734 | 30612 | 99.166 | 96.37 | D1624 | 64402 | 98.902 | 98.337 |
| D0735 | 39416 | 99.278 | 96.811 | D1625 | 29991 | 94.935 | 92.974 |
| D0736 | 38599 | 99.234 | 96.625 | D1626 | 54053 | 98.644 | 98.002 |
| D0737 | 40179 | 99.34 | 97.004 | D1627 | 58693 | 98.687 | 98.048 |
| D0738 | 27824 | 99.324 | 96.807 | D1628 | 42050 | 97.033 | 95.781 |
| D0739 | 64316 | 99.327 | 96.846 | D1629 | 50035 | 99.165 | 97.167 |
| D0740 | 66520 | 99.322 | 96.843 | D1630 | 27990 | 99.333 | 97.048 |
| D0741 | 30926 | 99.294 | 96.847 | D1631 | 46994 | 98.743 | 98.14 |
| D0742 | 63114 | 99.399 | 97.154 | D1632 | 48721 | 98.371 | 97.627 |
| D0743 | 53076 | 99.349 | 96.935 | D1633 | 42589 | 97.741 | 96.653 |
| D0744 | 70767 | 99.296 | 96.773 | D1634 | 25735 | 98.783 | 98.171 |
| D0745 | 33055 | 99.327 | 96.846 | D1635 | 15944 | 97.423 | 96.273 |
| D0746 | 59280 | 99.388 | 97.072 | D1636 | 50781 | 98.345 | 97.585 |
| D0747 | 54140 | 99.22 | 96.514 | D1637 | 24828 | 98.352 | 97.547 |
| D0748 | 60648 | 99.337 | 96.896 | D1638 | 62781 | 98.804 | 98.197 |
| D0749 | 39457 | 99.284 | 96.853 | D1639 | 42736 | 98.733 | 98.083 |
| D0750 | 90185 | 99.35 | 96.907 | D1640 | 28167 | 99.357 | 97.091 |
| D0751 | 44581 | 99.333 | 96.884 | D1641 | 40323 | 99.351 | 97.058 |
| D0752 | 66846 | 99.173 | 96.385 | D1642 | 71177 | 98.217 | 97.461 |
| D0753 | 34378 | 98.031 | 97.168 | D1643 | 31056 | 97.903 | 96.966 |
| D0754 | 70205 | 99.387 | 97.075 | D1644 | 65952 | 98.812 | 98.222 |
| D0755 | 62007 | 99.405 | 97.248 | D1645 | 41991 | 98.705 | 97.994 |
| D0756 | 44294 | 99.311 | 96.805 | D1646 | 31575 | 99.368 | 97.101 |
| D0757 | 38816 | 99.388 | 97.068 | D1647 | 52378 | 98.553 | 96.556 |
| D0758 | 37065 | 98.082 | 97.159 | D1648 | 60730 | 98.156 | 96.612 |
| D0759 | 41913 | 99.287 | 96.745 | D1649 | 47817 | 98.592 | 97.91 |
| D0760 | 55613 | 99.283 | 96.714 | D1650 | 33887 | 98.85 | 98.268 |
| D0761 | 64801 | 99.329 | 96.893 | D1651 | 33342 | 97.003 | 95.668 |
| D0762 | 81902 | 99.373 | 97.029 | D1652 | 47344 | 98.368 | 97.638 |
| D0763 | 59757 | 99.141 | 96.253 | D1653 | 25665 | 98.645 | 97.952 |
| D0764 | 59255 | 99.36 | 96.972 | D1654 | 39737 | 98.402 | 97.689 |
| D0765 | 37620 | 97.944 | 96.718 | D1655 | 61407 | 98.19 | 97.394 |
| D0766 | 57670 | 99.355 | 96.943 | D1656 | 30611 | 98.352 | 97.604 |
| D0767 | 32415 | 99.313 | 96.923 | D1657 | 49341 | 98.03 | 97.242 |
| D0768 | 37539 | 99.356 | 97.096 | D1658 | 53460 | 98.601 | 97.829 |
| D0769 | 67784 | 99.429 | 97.293 | D1659 | 34133 | 99.286 | 96.849 |
| D0770 | 63117 | 99.365 | 97.023 | D1660 | 43755 | 98.749 | 98.138 |
| D0771 | 72708 | 99.422 | 97.245 | D1661 | 38210 | 97.908 | 96.999 |
| D0772 | 62488 | 99.375 | 97.049 | D1662 | 65375 | 97.557 | 96.6 |
| D0773 | 39710 | 98.253 | 97.394 | D1663 | 43334 | 98.846 | 98.251 |
| D0774 | 69087 | 99.395 | 97.13 | D1664 | 56911 | 98.587 | 97.847 |
| D0775 | 61147 | 99.306 | 96.833 | D1665 | 54244 | 98.667 | 96.907 |
| D0776 | 68259 | 99.403 | 97.156 | D1666 | 26189 | 98.625 | 97.929 |
| D0777 | 73165 | 99.386 | 97.095 | D1667 | 45863 | 98.592 | 97.866 |
| D0778 | 42181 | 98.013 | 97.084 | D1668 | 49596 | 98.633 | 97.993 |
| D0779 | 30749 | 99.375 | 97.02 | D1669 | 58584 | 97.972 | 97.085 |
| D0780 | 43035 | 99.408 | 97.253 | D1670 | 33103 | 97.49 | 96.43 |
| D0781 | 32089 | 97.745 | 96.537 | D1671 | 38611 | 98.321 | 97.54 |
| D0782 | 49190 | 99.35 | 97.008 | D1672 | 52765 | 99.138 | 97.474 |
| D0783 | 77808 | 99.384 | 97.052 | D1673 | 26680 | 98.585 | 97.937 |
| D0784 | 45218 | 99.315 | 96.846 | D1674 | 62369 | 97.678 | 96.704 |
| D0785 | 35147 | 97.585 | 96.451 | D1675 | 42690 | 98.322 | 97.447 |
| D0786 | 43711 | 97.657 | 96.574 | D1676 | 24395 | 99.25 | 96.674 |
| D0787 | 63268 | 99.406 | 97.142 | D1677 | 24817 | 97.534 | 96.179 |
| D0788 | 34184 | 95.981 | 94.132 | D1678 | 34364 | 98.88 | 98.255 |
| D0789 | 63621 | 99.381 | 97.066 | D1679 | 51626 | 99.041 | 97.117 |
| D0790 | 81593 | 99.399 | 97.176 | D1680 | 57424 | 99.083 | 97.226 |
| D0791 | 55301 | 99.15 | 96.305 | D1681 | 21285 | 99.25 | 96.682 |
| D0792 | 35930 | 99.311 | 96.911 | D1682 | 27413 | 97.453 | 96.043 |
| D0793 | 68383 | 99.296 | 96.725 | D1683 | 43680 | 97.806 | 96.675 |
| D0794 | 69323 | 99.357 | 96.915 | D1684 | 28283 | 98.394 | 97.647 |
| D0795 | 57954 | 97.812 | 96.374 | D1685 | 45050 | 98.593 | 97.905 |
| D0796 | 30248 | 99.383 | 97.066 | D1686 | 37414 | 96.711 | 95.273 |
| D0797 | 69067 | 99.249 | 96.552 | D1687 | 79160 | 98.637 | 97.876 |
| D0798 | 66539 | 99.383 | 97.056 | D1688 | 29112 | 99.382 | 97.197 |
| D0799 | 203616 | 97.833 | 96.899 | D1689 | 15331 | 97.429 | 96.324 |
| D0800 | 39978 | 99.371 | 97.137 | D1690 | 40975 | 95.715 | 93.804 |
| D0801 | 35507 | 99.379 | 97.029 | D1691 | 43897 | 97.598 | 96.585 |
| D0802 | 44403 | 97.881 | 96.932 | D1692 | 69146 | 98.12 | 96.642 |
| D0803 | 33032 | 99.409 | 97.223 | D1693 | 35542 | 98.7 | 98.044 |
| D0804 | 58296 | 99.314 | 96.824 | D1694 | 30102 | 97.225 | 95.607 |
| D0805 | 35111 | 99.32 | 96.946 | D1695 | 47648 | 98.689 | 98.032 |
| D0806 | 57191 | 99.351 | 96.979 | D1696 | 40471 | 98.168 | 97.32 |
| D0807 | 36012 | 99.268 | 96.809 | D1697 | 53767 | 98.034 | 97.021 |
| D0808 | 30642 | 99.15 | 96.371 | D1698 | 47443 | 98.07 | 97.169 |
| D0809 | 36969 | 97.852 | 96.764 | D1699 | 43068 | 96.695 | 95.092 |
| D0810 | 38040 | 99.299 | 96.897 | D1700 | 51272 | 98.872 | 97.015 |
| D0811 | 36755 | 99.262 | 96.783 | D1701 | 53114 | 97.509 | 96.476 |
| D0812 | 39043 | 99.376 | 97.15 | D1702 | 36877 | 96.824 | 95.322 |
| D0813 | 41593 | 99.229 | 96.674 | D1703 | 47349 | 98.83 | 98.251 |
| D0814 | 80012 | 99.316 | 96.86 | D1704 | 52721 | 97.354 | 95.586 |
| D0815 | 66961 | 99.402 | 97.126 | D1705 | 38123 | 98.848 | 98.291 |
| D0816 | 71397 | 99.369 | 97.032 | D1706 | 69285 | 96.681 | 95.37 |
| D0817 | 36874 | 99.29 | 96.838 | D1707 | 59380 | 98.679 | 98.04 |
| D0818 | 34466 | 98.362 | 97.601 | D1708 | 30017 | 97.772 | 96.892 |
| D0819 | 29076 | 99.314 | 96.908 | D1709 | 71130 | 97.25 | 96.091 |
| D0820 | 57713 | 99.306 | 96.784 | D1710 | 45497 | 98.674 | 98.054 |
| D0821 | 30398 | 99.341 | 97.029 | D1711 | 32046 | 97.813 | 96.845 |
| D0822 | 34171 | 98.792 | 98.092 | D1712 | 45812 | 98.594 | 97.934 |
| D0823 | 11326 | 97.329 | 95.976 | D1713 | 44097 | 97.919 | 97.079 |
| D0824 | 34938 | 99.297 | 96.885 | D1714 | 71325 | 98.477 | 97.746 |
| D0825 | 13361 | 97.391 | 96.138 | D1715 | 28571 | 97.894 | 96.803 |
| D0826 | 44378 | 97.485 | 96.451 | D1716 | 74150 | 98.441 | 97.648 |
| D0827 | 62564 | 99.425 | 97.228 | D1717 | 45745 | 98.83 | 98.268 |
| D0828 | 37403 | 97.743 | 96.672 | D1718 | 38789 | 98.786 | 98.206 |
| D0829 | 54627 | 99.298 | 96.745 | D1719 | 46277 | 98.635 | 98.001 |
| D0830 | 42939 | 99.419 | 97.315 | D1720 | 24072 | 98.243 | 97.386 |
| D0831 | 43774 | 99.341 | 97.002 | D1721 | 53074 | 98.792 | 98.182 |
| D0832 | 32421 | 98.737 | 98.058 | D1722 | 64728 | 98.481 | 97.773 |
| D0833 | 38022 | 99.429 | 97.358 | D1723 | 59246 | 98.74 | 98.15 |
| D0834 | 66453 | 96.953 | 94.659 | D1724 | 54454 | 98.679 | 98.039 |
| D0835 | 44783 | 99.361 | 97.051 | D1725 | 73033 | 97.656 | 96.711 |
| D0836 | 33990 | 99.346 | 97.022 | D1726 | 35313 | 98.225 | 97.373 |
| D0837 | 36599 | 98.476 | 97.702 | D1727 | 58454 | 99.13 | 97.471 |
| D0838 | 43564 | 96.636 | 95.08 | D1728 | 58311 | 98.671 | 98.053 |
| D0839 | 57889 | 99.403 | 97.219 | D1729 | 41725 | 97.991 | 97.206 |
| D0840 | 65610 | 99.321 | 96.833 | D1730 | 40861 | 99.289 | 96.84 |
| D0841 | 78852 | 99.351 | 96.976 | D1731 | 57264 | 98.635 | 97.964 |
| D0842 | 31849 | 99.282 | 96.797 | D1732 | 46957 | 98.218 | 97.212 |
| D0843 | 70853 | 99.37 | 96.974 | D1733 | 45969 | 98.072 | 97.234 |
| D0844 | 34810 | 97.562 | 96.312 | D1734 | 65054 | 98.207 | 97.443 |
| D0845 | 72946 | 99.375 | 97.041 | D1735 | 42437 | 98.771 | 98.199 |
| D0846 | 47941 | 98.154 | 97.145 | D1736 | 27853 | 98.711 | 98.049 |
| D0847 | 58392 | 99.378 | 97.037 | D1737 | 48574 | 97.476 | 96.365 |
| D0848 | 38274 | 98.683 | 97.973 | D1738 | 10726 | 97.312 | 95.974 |
| D0849 | 42154 | 99.314 | 96.887 | D1739 | 58416 | 97.904 | 96.963 |
| D0850 | 40619 | 97.564 | 96.479 | D1740 | 35280 | 99.219 | 96.623 |
| D0851 | 39989 | 99.283 | 96.849 | D1741 | 27275 | 98.843 | 98.245 |
| D0852 | 59963 | 99.037 | 95.944 | D1742 | 66493 | 98.634 | 97.789 |
| D0853 | 45418 | 99.36 | 97.097 | D1743 | 58802 | 98.808 | 98.223 |
| D0854 | 73241 | 97.97 | 96.884 | D1744 | 35113 | 98.876 | 98.339 |
| D0855 | 36798 | 99.335 | 97.028 | D1745 | 60772 | 98.691 | 98.101 |
| D0856 | 25093 | 98.574 | 97.878 | D1746 | 73966 | 98.27 | 97.522 |
| D0857 | 52177 | 97.628 | 96.549 | D1747 | 39796 | 98.324 | 97.605 |
| D0858 | 53330 | 99.308 | 96.885 | D1748 | 36086 | 97.609 | 96.664 |
| D0859 | 71063 | 99.349 | 96.967 | D1749 | 49338 | 98.814 | 98.165 |
| D0860 | 38016 | 97.874 | 96.809 | D1750 | 26357 | 99.382 | 97.19 |
| D0861 | 30213 | 98.144 | 97.264 | D1751 | 54643 | 98.025 | 96.34 |
| D0862 | 36604 | 98.01 | 97.149 | D1752 | 37857 | 98.751 | 98.184 |
| D0863 | 64661 | 99.399 | 97.144 | D1753 | 27582 | 94.656 | 92.015 |
| D0864 | 41701 | 98.406 | 97.641 | D1754 | 25523 | 96.756 | 95.455 |
| D0865 | 35073 | 98.134 | 97.215 | D1755 | 35727 | 99.238 | 97.079 |
| D0866 | 36404 | 99.336 | 96.993 | D1756 | 60061 | 98.315 | 97.391 |
| D0867 | 34468 | 99.323 | 96.948 | D1757 | 55773 | 98.797 | 98.175 |
| D0868 | 31806 | 99.367 | 97.119 | D1758 | 35169 | 98.172 | 97.423 |
| D0869 | 66348 | 99.414 | 97.19 | D1759 | 32929 | 99.245 | 96.715 |
| D0870 | 37381 | 97.923 | 96.819 | D1760 | 69775 | 97.129 | 96.006 |
| D0871 | 44920 | 99.378 | 97.186 | D1761 | 43535 | 98.67 | 98.073 |
| D0872 | 44268 | 99.337 | 97.015 | D1762 | 58648 | 98.147 | 97.308 |
| D0873 | 41285 | 97.334 | 96.085 | D1763 | 27919 | 97.388 | 96.085 |
| D0874 | 30804 | 99.39 | 97.197 | D1764 | 32201 | 98.21 | 97.455 |
| D0875 | 76589 | 99.371 | 97.027 | D1765 | 84326 | 98.704 | 98.051 |
| D0876 | 30809 | 98.132 | 97.136 | D1766 | 29389 | 99.312 | 96.867 |
| D0877 | 48863 | 99.333 | 96.974 | D1767 | 52776 | 98.175 | 97.246 |
| D0878 | 71425 | 99.357 | 96.956 | D1768 | 28402 | 99.308 | 96.908 |
| D0879 | 37850 | 99.272 | 96.801 | D1769 | 23411 | 97.656 | 96.552 |
| D0880 | 42102 | 99.364 | 97.086 | D1770 | 40412 | 98.865 | 98.289 |
| D0881 | 53463 | 99.138 | 97.304 | D1771 | 46201 | 95.079 | 93.086 |
| D0882 | 58737 | 98.568 | 97.89 | D1772 | 34932 | 99.223 | 96.533 |
| D0883 | 40678 | 99.36 | 97.092 | D1773 | 53683 | 98.68 | 98.031 |
| D0884 | 41339 | 99.368 | 97.132 | D1774 | 49017 | 98.047 | 97.184 |
| D0885 | 36508 | 99.386 | 97.198 | D1775 | 72413 | 98.562 | 97.901 |
| D0886 | 40912 | 99.356 | 96.925 | D1776 | 31444 | 97.594 | 96.405 |
| D0887 | 39891 | 98.032 | 97.118 | D1777 | 30208 | 99.324 | 96.942 |
| D0888 | 34133 | 99.398 | 97.215 | D1778 | 58737 | 98.746 | 98.073 |
| D0889 | 51530 | 99.4 | 97.128 | D1779 | 32952 | 96.854 | 95.597 |
| D0890 | 42157 | 98.632 | 97.857 | D1780 | 75710 | 97.396 | 96.336 |

**Fig S1.** Flow diagram of participants’ selection for the analyses of present study.

**
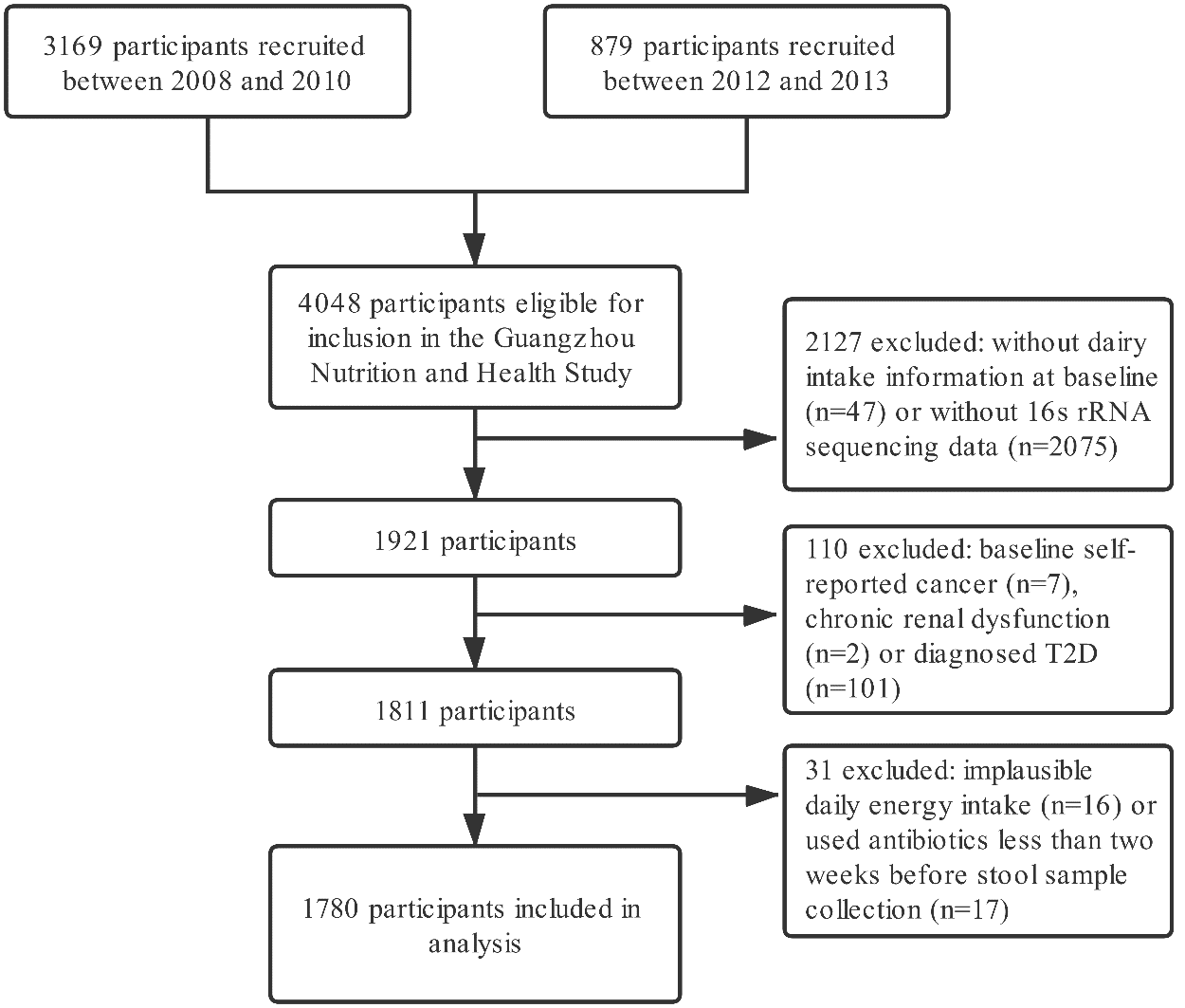
**

**Fig S2.** Forest plot of the interaction of dairy consumption with gut microbial features on blood triglycerides. Standardized differences were expressed as the difference in cardiometabolic risk factors (in SD unit) by 1 serving/d difference in each dairy product. Linear regression models were adjusted for age, sex, BMI, smoking status, drinking status, education attainment, household income, physical activity, total energy intake, dietary intakes of vegetables, fruit, fish, egg and red meat, and baseline triglycerides. **#**Associations were stratified by the median of the α diversity index (in SD unit). **P* < 0.05.

**
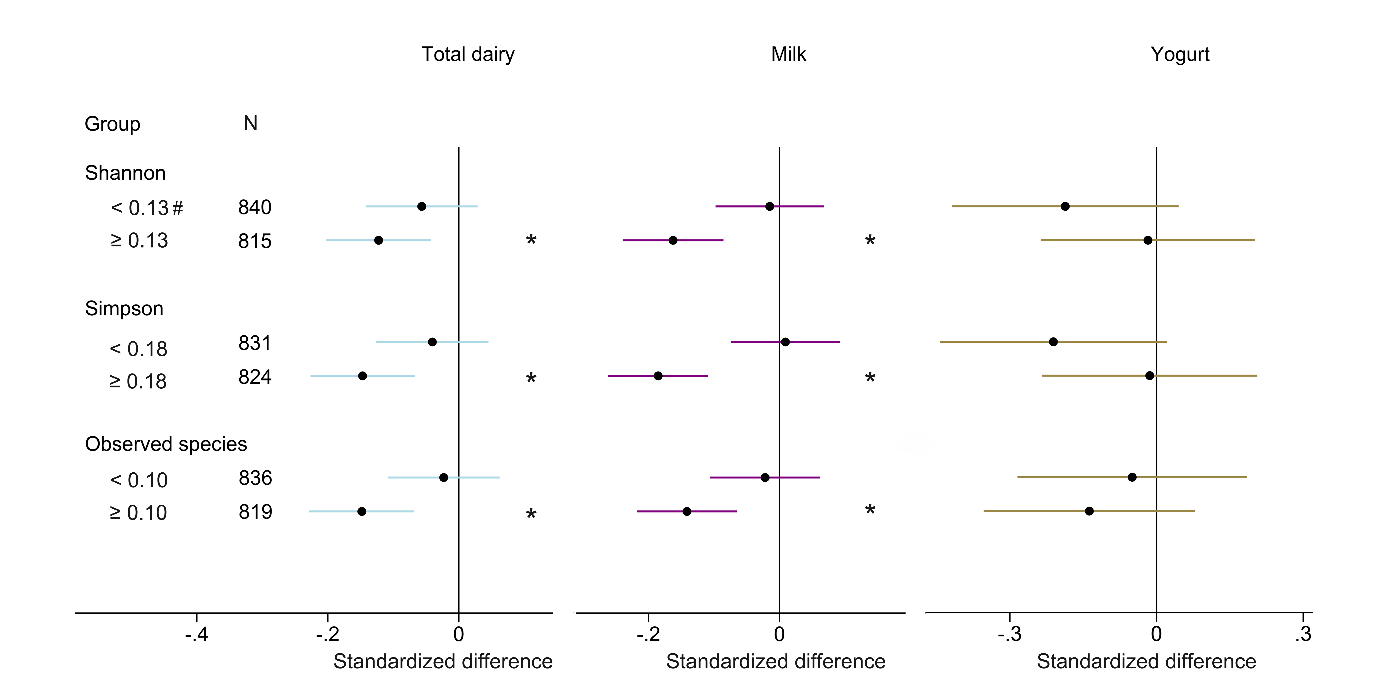
**
